# Tracing Carbon Sources through Aquatic and Terrestrial Food Webs Using Amino Acid Stable Isotope Fingerprinting

**DOI:** 10.1371/journal.pone.0073441

**Published:** 2013-09-17

**Authors:** Thomas Larsen, Marc Ventura, Nils Andersen, Diane M. O’Brien, Uwe Piatkowski, Matthew D. McCarthy

**Affiliations:** 1 Leibniz-Laboratory for Radiometric Dating and Stable Isotope Research, Christian-Albrechts Universität zu Kiel, Kiel, Germany; 2 Biogeodynamics and Biodiversity Group, Centre for Advanced Studies of Blanes, Spanish Research Council (CEAB-CSIC), Blanes, Catalonia, Spain; 3 Institut de Recerca de l’Aigua, Universitat de Barcelona, Barcelona, Catalonia, Spain; 4 Institute of Arctic Biology and Department of Biology and Wildlife, University of Alaska Fairbanks, Fairbanks, Alaska, United States of America; 5 GEOMAR, Helmholtz-Zentrum für Ozeanforschung Kiel, Kiel, Germany; 6 Ocean Sciences Department, University of California Santa Cruz, Santa Cruz, California, United States of America; University of Otago, New Zealand

## Abstract

Tracing the origin of nutrients is a fundamental goal of food web research but methodological issues associated with current research techniques such as using stable isotope ratios of bulk tissue can lead to confounding results. We investigated whether naturally occurring δ^13^C patterns among amino acids (δ^13^C_AA_) could distinguish between multiple aquatic and terrestrial primary production sources. We found that δ^13^C_AA_ patterns in contrast to bulk δ^13^C values distinguished between carbon derived from algae, seagrass, terrestrial plants, bacteria and fungi. Furthermore, we showed for two aquatic producers that their δ^13^C_AA_ patterns were largely unaffected by different environmental conditions despite substantial shifts in bulk δ^13^C values. The potential of assessing the major carbon sources at the base of the food web was demonstrated for freshwater, pelagic, and estuarine consumers; consumer δ^13^C patterns of essential amino acids largely matched those of the dominant primary producers in each system. Since amino acids make up about half of organismal carbon, source diagnostic isotope fingerprints can be used as a new complementary approach to overcome some of the limitations of variable source bulk isotope values commonly encountered in estuarine areas and other complex environments with mixed aquatic and terrestrial inputs.

## Introduction

During the last 30 years, stable isotope analysis has emerged as one of most powerful tools for tracing organic carbon in food webs. Analyses of total organic matter (“bulk”) have become widespread due to the relative ease and low cost of sample preparation and analysis. However, the potential of variable environmental conditions to influence carbon isotopic ratios at the base of the food web (δ^13^C) is a serious drawback for disentangling aquatic and terrestrial sources [1], [2], [3], [4]. With the advent of new continuous flow technologies, compound specific isotope analysis is increasingly employed as a complementary tool for food web analysis. Isotope analysis of fatty acids in conjunction with their structural compositions are now widely used to investigate biosynthetic sources since their molecular structure are tied to their biosynthetic origins (e.g. [5]). However, fatty acids account for only a small fraction of total organic carbon fluxes, and they tend to undergo degradation and transformation during food web passage [6]. Amino acids (AAs), in contrast, account for the large majority of organic nitrogen, and about half of total carbon in most organisms [7] and therefore are among the major conduits of carbon through food chains. The main methodological drawback of the 20 protein AAs is that they are ubiquitous in all life forms. However, about half of the AAs can only be synthesized by bacteria, fungi and photoautotrophs, and are therefore essential or indispensable for animal diets. Since these essential AAs (EAAs) typically pass from food source to consumer without alteration to their carbon skeletons [8], [9], a method for tracking their origins and fluxes could greatly advance our understanding of nutrient cycling and trophic relationships.

Recent research has shown that naturally occurring δ^13^C_AA_ patterns contain information of both biosynthetic origin and mode of carbon acquisition [10], and that the EAA group is particularly diagnostic of origin [11]. These δ^13^C_AA_ patterns represent the sum of the isotopic fractionations associated with the individual biosynthetic pathways and associated branch points for each AA (e.g. [12]). Larsen et al. [11] found that the δ^13^C_AA_ patterns of terrestrial plants, bacteria and fungi were distinct and consistent, and proposed these “stable isotope fingerprints” as a tool for tracing sources of organic matter in terrestrial ecosystems. Comparisons of δ^13^C_EAA_ patterns between laboratory reared consumers and known diets have also indicated that stable isotope fingerprints are passed on to consumers [11], [13], [14]. In spite of these advances, it has until now been unresolved whether δ^13^C_AA_ patterns can distinguish between aquatic and terrestrial primary producers [14] and to what extent variable growth conditions for aquatic producers may influence δ^13^C_AA_ patterns. The factors that affect bulk and compound specific isotope patterns are fundamentally different. While bulk δ^13^C values for a given producer largely are determined by the ratio of carbon fixation to carbon flux into the cell [15], δ^13^C_AA_ patterns are determined downstream of the Calvin cycle by AA biosynthetic pathways and associated branching points in the central metabolism. For bulk δ^13^C based studies, temporal and spatial variations of inorganic carbon sources and other environmental conditions therefore pose a challenge [16], [17]; particularly in the highly productive coastal benthic and estuarine habitats that are also the zones of most organic carbon deposition in modern biogeochemical cycles. A measure based on compound specific stable isotope analysis that could distinguish between terrestrial and aquatic sources of organic matter across varying ecological conditions would therefore have profound implications for biological research across a number of disciplines.

Here we investigated whether δ^13^C_AA_ patterns based approaches could transcend some of the limitations associated with bulk δ^13^C, by characterizing δ^13^C_AA_ values for a large set of different algae and vascular plants. We tested whether these two groups have different δ^13^C_AA_ patterns, and compared these with δ^13^C_AA_ patterns from heterotrophic bacteria and fungi. Further, to test the potential for δ^13^C_AA_ source information to be independent of variation in bulk δ^13^C values, we analyzed two marine primary producers sampled from a range of different environments within their natural habitats. Finally, to assess the practical relevance of using δ^13^C_AA_ patterns as diagnostic and quantitative biomarkers in actual ecosystems we analyzed consumers from freshwater, pelagic, and estuarine systems.

## Materials and Methods

### Sampling Design

To test whether freshwater, marine and terrestrial primary producers have different δ^13^C_AA_ patterns, we collected and cultured samples from wide variety of primary producers (micro- and macroalgae, and terrestrial plants). In the field, we only collected fresh and newly emerged thallus and leaves. For microbial reference samples we obtained axenically cultured bacteria and fungi. For testing the potential for δ^13^C_AA_ source information to be independent of variation in bulk δ^13^C values among aquatic producers, we acquired multiple samples of the seagrass *Posidonia oceanica* and the giant kelp *Macrocystis pyrifera* collected from a range of different environments within their natural habitats. For *P. oceanica,* the maximum photosynthetically active radiation above their canopies ranged from 50 to 310 mmol m^−2^ s^−1^ between the sampling locations and the coverage of leaf necrosis ranged from 0 to 37.5%. *M. pyrifera* samples were collected either in spring or late fall, periods of contrasting ocean conditions that result in widely divergent bulk isotopic values [18]. Note that our sampling was not designed for investigating the influence of geographical region on terrestrial plants since a previous study [14] found no systematic differences in δ^13^C_AA_ patterns from greenhouse plants and those collected in boreal and mangrove ecosystems. Finally, to assess the practical relevance of using δ^13^C_AA_ patterns as diagnostic biomarkers in actual ecosystems we analyzed consumers from three well-studied ecosystems: from a marine pelagic ecosystem in the central North Pacific Ocean the carnivorous fish species opah (*Lampris guttatus)*, common dolphinfish (*Coryphaena hippurus)* and broadbill swordfish (*Xiphias gladius*), from a littoral marine system (estuarine) the California mussel (*Mytilus californianus*), and from oligotrophic arctic lakes the water flea *Daphinia* sp. and seston.

### Sample Acquisition and Preparation

A detailed list of all our field samples and their locations is provided in Table S1. Here follows a general description of sampling locations, protocols and permits. All macroalgae (22 species), two seagrasses and two mussels were collected by the Californian shore. The macroalgae except *M. pyrifera* (see below) were collected on state tidelands for which collection without a permit is allowed for less than ten pounds fresh weight. The seagrass samples were provided under a permit to Joseph M. Long Marine Laboratory, Santa Cruz. Subsamples of mussels were obtained from recreationally harvested mussels under a permit to Natasha Vokhshoori. The three pelagic fish samples were collected as in Choy et al. [19] with NOAA longline observers, and locations for the fish samples are approximate and are reported as the centers of 5×5 degree cells in accordance with NOAA confidentiality policies. Soils and the majority of terrestrial plants (10 out of 12 species) were collected adjacent to five Alaskan tundra lakes in which we also collected seston (5–80 µm size particles) and *Daphnia*. The Alaskan samples were collected on land owned by the Alaskan state or Bureau of Land Management where no permission was required because we sampled on day trips by foot or rafts leaving no permanent marks; soil samples were collected between 2 and 8 cm below the soil surface and amounted to <50 g dry weight for each location; each plant sample amounted <1 g dry weight. No permit was required for sacrificing *Daphnia,* which are not protected or endangered; they were sampled with a zooplankton net (150–200 µm) and kept alive until sorting at Toolik Field Station. The terrestrial plant collection was supplemented with two species from public owned land in Denmark where no permission was required for sampling. We obtained five *P. oceanica* samples from the Catalonian shore in the Mediterranean Sea under a permit to Teresa Alcoverro by the Catalan Water Agency, and the five *M. pyrifera* samples from the Californian shore were sampled under a permit to Melissa M. Foley [18] by NOAA’s Monterey Bay National Marine Sanctuary. All field samples were kept on crushed ice in coolers and stored between 1 and 3 days before thoroughly washing them in milliQ water (except for animal and seston samples). Seagrass leaves were further cleaned by scraping off epiphytes with a razor blade. The seston samples that during sampling had been collected into 500 ml bottles were in the laboratory concentrated onto GF/C glass microfiber filters (Whatman). For analysis of animal samples, we prepared whole tissue samples of *Daphnia* and muscle tissue for mussels and fish. All samples were dried at 50°C except for animal, seston and soil samples, which were freeze-dried. After drying the samples were homogenized with a mortar.

Microalgae comprised of five diatoms, two cyanobacteria, two chlorophytes, three haptophytes, and two crysophytes obtained from existing axenic cultures at GEOMAR, Kiel, Germany or the culture collection of algae of Goettingen University, Germany. See Table S2 for detailed list of laboratory samples. The microalgae were cultured between April 2010 and January 2011 at GEOMAR in sterile 225 cm^2^ tissue culture flasks with vented cap in brackish water (13.9 psu) collected from Kiel Fjord or seawater (31.2 psu) collected at Multimar-Wattforum by the German North Sea. Water mixed with added nutrients was sterile filtered (Whatman celluloseacetate 0.2 µm filter) (see Table S2 for light, temperature and nutrient regimes). In addition we collected three non-sterile filtered water samples from Kiel Fjord February 2012 for the use of culturing of natural assemblages of microalgae in culture tanks. Visual inspection of these samples revealed between 60–80% dominance of diatoms. All microalgae were harvested after 5 to 21 days during exponential growth or right after the onset of the lag phase on either a Durapore PVDF 0.22 µm pore size filter (Sigma-Aldrich, Germany), GF/C glass microfiber filters (Whatman) or by centrifugation in 50 ml vials at 3300 g. The algae were subsequently freeze dried, and surplus substrates with live cultures were autoclaved.

Laboratory grown fungi and bacteria samples were either obtained from a previous study [11] or isolated and cultured at the Institute of Arctic Biology, University of Alaska Fairbanks from soil or water samples collected during the Alaskan field sampling mentioned above (see Table S2 for details). For isolating fungal and bacterial strains, the growth media were treated with either antibiotics (Streptomycin and Tetracycline hydrochloride) or fungicides (Amphotericin and Nystatin, all chemicals were from Sigma-Aldrich, St. Louis, Missouri, USA). After isolation under sterile conditions, all bacteria samples were grown on solid media (Bacto Agar BD, Sparks, Maryland, USA) and fungi were grown in liquid media flasks in a shaking incubator (Innova 4230, New Brunswick Scientific, Edison, New Jersey, USA) and harvested after one to three weeks. We used amino acid free nutrient media containing 15 g L^−1^ of one of the following nutrient mixes ‘Czapek’, ‘MAG’ and ‘MMN’ (See Table S2 for temperature and nutrient regimes). Microbial samples in liquid media were harvested by centrifugation in 50 ml tubes (2200 g) and freeze dried after harvesting. All substrates with viable cultures were autoclaved after harvesting.

### Elemental and Isotope Analysis

Elemental content and bulk isotope ratios of plants, bacteria, fungi, macroalgae and animals were measured at the UCSC Stable Light Isotope Facility. Approximately 1 mg of sample was pelletized into tin capsules and analyzed on a Carlo Erba 1108 linked to a Thermo Finningan DeltaPlus XP mass spectrometer with an analytical standard deviation of typically<±0.15‰ (n = 3). Elemental content and bulk isotope ratios of microalgae were determined on 2 mg samples pelletized into tin capsules at the UC Davis Stable Isotope Facility using a PDZ Europa ANCA-GSL elemental analyzer interfaced to a PDZ Europa 20–20 isotope ratio mass spectrometer (Sercon Ltd., Cheshire, UK). Isotope data are expressed in delta (δ) notation as ((R_sample_/R_standard_) –1) × 1000‰, where R is the ratio of heavy to light isotope; and the standard is Vienna Pee Dee Belemnite (VPDB) for carbon and air for nitrogen.

For δ^13^C analysis of individual AAs (δ^13^C_AA_) we transferred between 1.5 and 7 mg of sample to Pyrex culture tubes (13×100 mm). Samples were flushed with N_2_ gas, sealed, and hydrolyzed in 1–2 ml 6 N HCl (37% HCl diluted with Milli-Q water, Merck, Darmstadt, Germany) at 110°C in a heating block for 20 h. After hydrolysis, samples collected on GF/C filters and coralline algae were purified with Dowex 50WX8 cation exchange resin according to Amelung & Zhang [20] and He et al [21]. In the remaining samples we removed lipophilic compounds by adding 2 ml *n*-hexane/DCM (6∶5, *v*/*v*) to the Pyrex tubes that were flushed shortly with N_2_ gas and sealed before vortexing for 30 s. The aqueous phase was then filtered through a Pasteur pipette lined with glass wool that had been pretreated at 450°C. All samples were transferred into 4 ml dram vials before evaporating the samples to dryness under a steam of N_2_ gas for 30 minutes at 110°C in a heating block. The samples were stored at −18°C. To volatize the AAs, we followed the derivatization procedure by Corr et al [22] methylating the dried samples with acidified methanol and subsequently acetylating them with a mixture of acetic anhydride, triethylamine and acetone (NACME: *N*-acetyl methyl ester derivatives). As a precautionary measure to reduce oxidation of amino acids during derivatization, we flushed and sealed reaction vials with N_2_ gas prior to the methylation and acetylation reactions. To account for carbon added during derivatization [23] and variability of isotope fractionation during analysis, we also derivatized and analyzed pure amino acids with known δ^13^C values. Nor-leucine was used as an internal standard. Amino acid derivatives were injected with an autosampler into an Agilent Single Taper Ultra Inert Liner (#5190-2293) that was held at 280°C for 2 min. The compounds were separated on a Thermo TraceGOLD TG-200MS GC column (60 m×0.32 mm×0.25 um) installed on an Agilent 6890N gas chromatograph (GC). The oven temperature of the GC started at 50°C and heated at 15°C min^−1^ to 140°C, followed by 3°C min^−1^ to 152°C and held for 4 min, then 10°C min^−1^ to 245°C and held for 10 min, and finally 5°C min^−1^ to 290°C and held for 5 min. The GC was interfaced with a MAT 253 isotope ratio mass spectrometer (IRMS) via a GC-III combustion (C) interface (Thermo-Finnigan Corporation). All samples were analyzed in triplicate. The average reproducibility for the internal standard nor-leucine (Nle) was ±0.4‰ (n = 3) and the amino acid standards ranged from ±0.1‰ for Phe to ±0.6‰ for Thr (n = 12–15 for each batch). Of the amino acids we were able to analyze (see Fig. S1 for GC-C-IRMS chromatogram) the following were defined as non-essential for animals: alanine (Ala), asparagine/aspartic acid (Asx), glutamine/glutamic acid (Glx), glycine (Gly), and tyrosine (Tyr). The following were defined as essential: histidine (His), isoleucine (Ile), leucine (Leu), lysine (Lys), methionine (Met), phenylalanine (Phe), threonine (Thr), and valine (Val).

### Calculations and Statistical Analyses

All statistical analyses except the mixing modeling were performed in R version 2.12.1 [24] with RStudio interface version 0.96.330. All values in the text are given as mean ± standard deviation. To explore patterns and group memberships in our dataset we performed Ward’s hierarchical clustering (R-package cluster) and principal component analysis (PCA, R-package vegan) on δ^13^C values of amino acids that had been normalized to their respective sample means denoted as δ^13^C_AAn_. Prior to applying statistical analysis the data were tested for univariate normality by visually checking whether there were departures from normality on Q-Q plots. His and Met were excluded from the analyses due to missing measurements caused by concentrations below detection limits. Differences in each amino acid between different producer groups were tested with ANOVA with Tukey HSD post-hoc tests. To examine combinations of independent variables (i.e. δ^13^C_AA_ values) that best explained differences between the categorical variables (i.e. the groups defined by the PCA and one-way ANOVA tests) and to construct models for predicting membership of unknown samples, we performed linear discriminant function analysis (LDA, R package MASS [25]) on δ^13^C_AA_ values. For calculating the probability of group membership of the classifier samples we used a leave-one-out cross-validation approach. To test the null hypothesis that there was no difference in classification among the groups we applied Pillai’s trace (MANOVA). Relative contributions of EAAs from diets to consumers was estimated in the software FRUITS (version 0.1, http://sourceforge.net/projects/fruits) [26] with normalized isotope values. FRUITS also considers the biochemical composition of sources and which sources are most likely to contribute the most. FRUITS is executed with BUGS, which is a software package for performing “Bayesian inference Using Gibbs Sampling” that includes an expert system for determining an appropriate Markov chain Monte Carlo scheme based on the Gibbs sampling.

## Results

**Figure 1:**
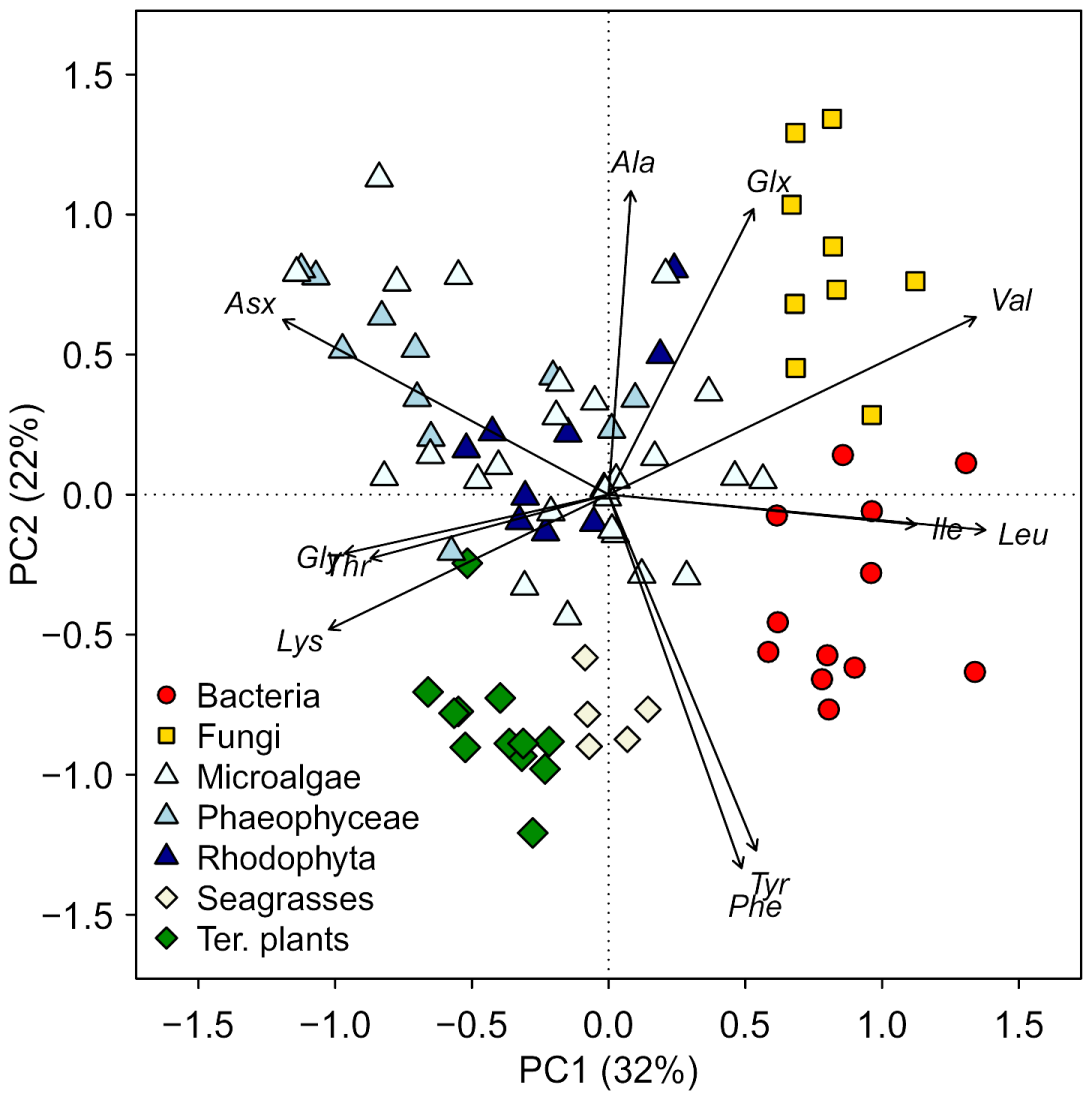
The principal component analysis of δ^13^C_AAn_ values of different producers show a range of different isotope patterns between bacteria, fungi, vascular plant and algae. None of the microalgal or macroalgal group clustered separately from one another. Values in parentheses are the percentage variation accounted by each axes. The first axis separates the photoautotrophs from the microbes, and the second axis separates vascular plants from algae, and fungi from bacteria. The fairly similar vector lengths show that almost all amino acids were important for the variations of the two first ordination components. See Table S4 for analytical details.

To assess differences in δ^13^C_AA_ patterns we first carried out a PCA with δ^13^C_AAn_ values that in contrast to a LDA does not require categorical variables. With this analysis we found that the samples clustered according to their major phylogenetic associations: algae, bacteria, fungi and vascular plants (Fig. 1). The first principal component accounted for 33% of the variation, and separated the photoautotrophs from the microorganisms. The second principal component accounted for 27% of the variation and separated algae from vascular plants, and fungi from bacteria. The AAs in the PCA grouped largely according to their biosynthetic families, with the exception that Ile grouped with the pyruvate AAs (Ala, Leu and Val), but biosynthetically belongs to the oxaloacetate family (Asx, Lys and Thr). The third group consisted of the aromatic AAs, Tyr and Phe. See Table S3 for the δ^13^C values for each AA measured, and Table S4 for detailed PCA results. We then tested which of the δ^13^C_AAn_ values were significantly different between algae, terrestrial plants, bacteria and fungi with ANOVA (Table S5). Seven amino acids (Ala, Phe, Tyr, Thr, Val, Leu and Lys) were significantly different between algae and terrestrial plants, all AAs except Glx were significantly different between algae and bacteria, and six (Glx, Gly, Ile, Leu, Lys and Val) were significantly different between algae and fungi (see Table S5 for direction of the differences). The microalgal groups (chlorophytes, chrysophytes, cyanobacteria, diatoms, and haptophytes) only displayed subtle differences between δ^13^C_AAn_ values (Table S5). For this reason we grouped them to a single group and tested them against the two macroalgal groups. We found the most notable difference between brown algae and microalgae with five significantly different AAs (Ala, Asx, Ile, Lys, Tyr).

**Figure 2:**
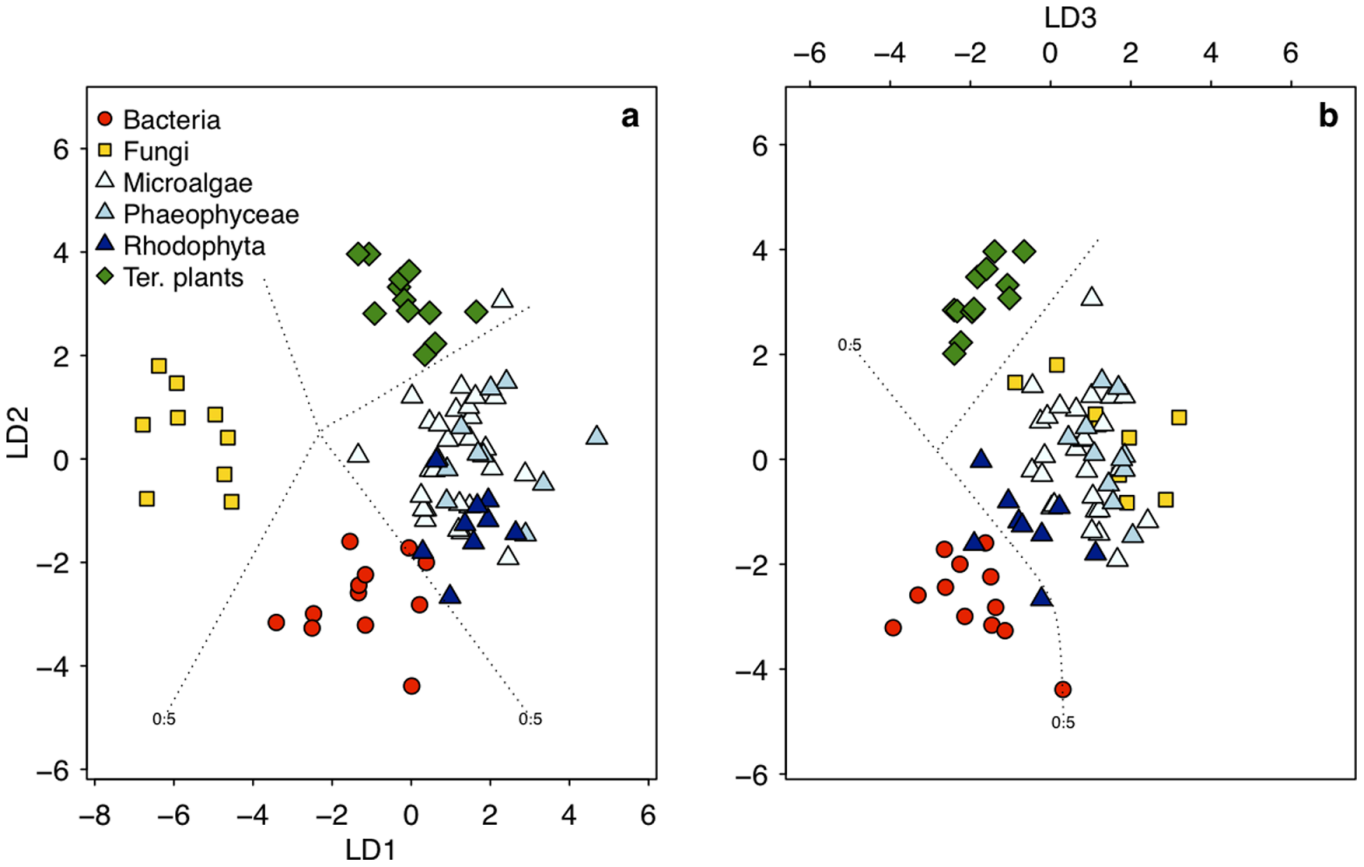
Linear discriminant function analysis based on the δ^13^C_EAA_values (Ile, Leu, Lys, Phe, Thr, Val) of bacteria, fungi, algae and terrestrial plants. In the left figure (a) displaying the scores of the first two discriminant axes, fungi and terrestrial plants each cluster separately from algae and bacteria. In the right figure (b) displaying the second and third discriminant axes, bacteria are separated apart from the algae, fungi and terrestrial plants. The dotted lines represent confidence ranges at P = 0.5. See Table S6 for details.

**Figure 3:**
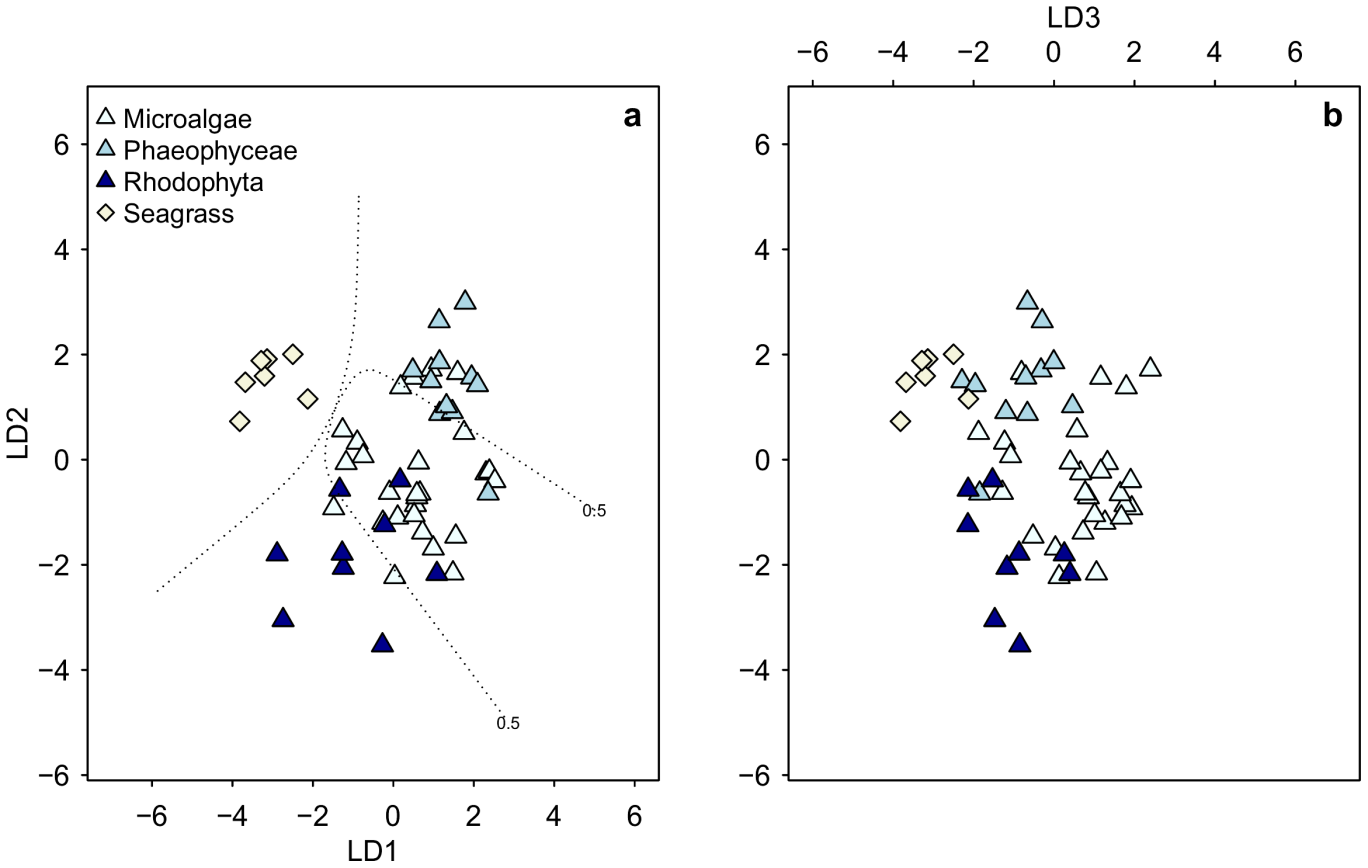
Linear discriminant function analysis with the δ^13^C_EAA_values (Ile, Leu, Lys, Phe, Val) from the three algal groups. It separates all seagrass samples from the three algal groups. The majority of the algal samples classified correctly within their own groups (Table S7). The dotted lines represent confidence ranges at P = 0.5; confidence ranges are only displayed in the left figure (a) because the third linear discriminant in right figure (b) only explained 14%.

We then applied LDA to our δ^13^C_EAA_ data to identify which of the six EAAs (Ile, Leu, Lys, Phe, Thr, Val) were most important for distinguishing between algae, bacteria, fungi and terrestrial plants. The seagrass samples were omitted from this analysis due to a small number of samples (the number of samples did not exceed the number of EAAs). Bacterial, fungal, and terrestrial plant samples classified with 99.1±2.6% posterior probability within their own groups (Fig. 2, Table S6). The microalgal samples classified with 97.3±8.1% probability as algae with *Melosira varians* and *Isochrysis galbana* having the lowest probabilities (84% and 61%, respectively). All brown algae samples (Phaeophyceae) classified with 100% probability as algae, and eight out of nine red algae (Rhodophyta) classified with 98.6±3.8% probability as algae. The ninth red algae, *Osmundea spectabilis*, classified as a bacterium (54% probability) rather than an alga (46% probability). The most important linear discriminants for separating the four categorical variables were Lys, Phe, Leu and Val. We created a second LDA model based on the five most informative EAAs (Ile, Leu, Lys, Phe, Val) to assess to what extent it would be possible to separate the three algal groups (microalgae, brown algae and red algae) and seagrass samples from each other. Of all 27 microalgal samples, 24 samples classified as microalgae, 10 out of 12 brown algae classified as brown algae, 7 out of 9 red algae classified as red algae, and all 7 seagrass samples classified as seagrass (Fig. 3, Table S7). None of the algal samples classified as seagrass.

**Figure 4:**
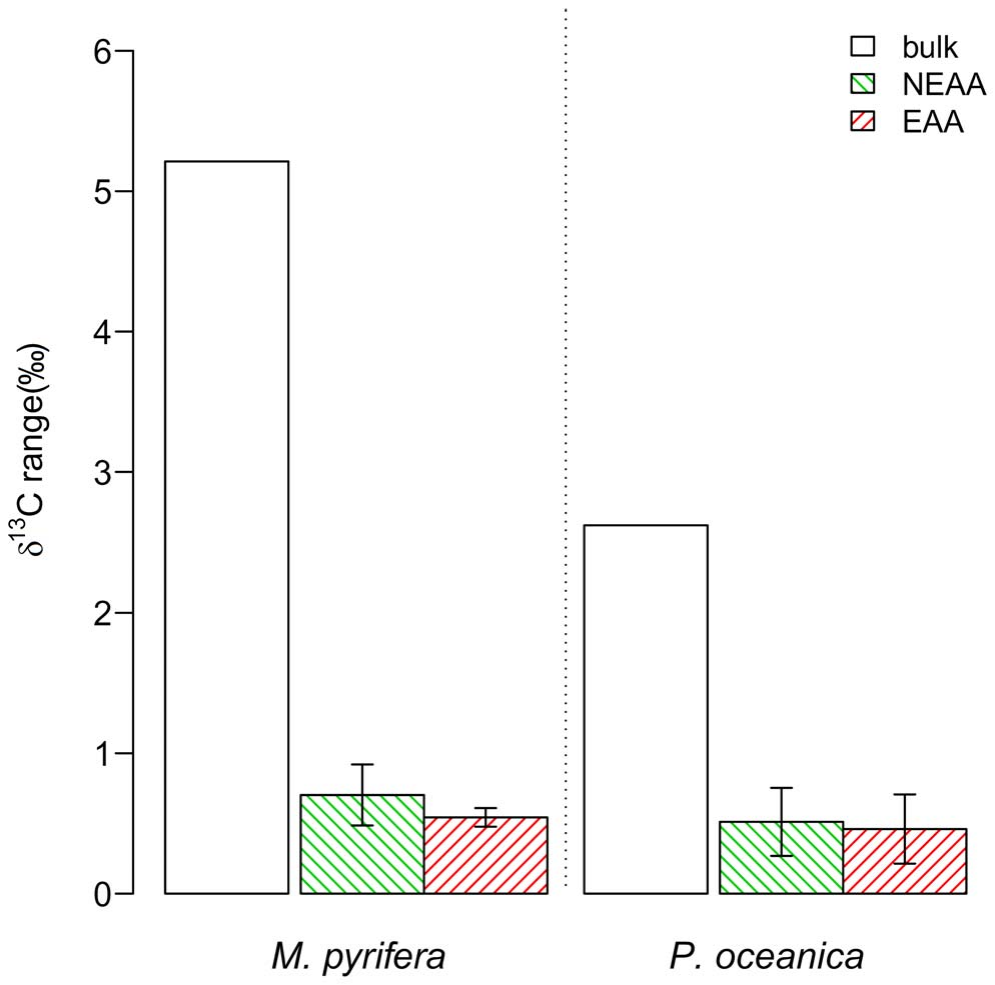
Bars representing the maximum range for individual amino acid δ^13^C_n_ values (normalized to their means) and bulk δ^13^C values across five giant kelp samples (*Macrocystis pyrifera*) or five seagrass samples (*Posidonia oceanica*). The bars for the amino acids represent the mean and standard deviations of either five non-essential (NEAA) or six essential (EAA) amino acids.

The second major question asked by our study was to what extent δ^13^C_AA_ patterns are affected by environmental conditions. To answer this question we analyzed seagrass (*P. oceanica*) and giant kelp samples (*M. pyrifera*) [16] across a variety of growth conditions (see Table S1 for details). For both species the range in δ^13^C values was five- to ten-fold greater for bulk than δ^13^C_AAn_ values (Fig. 4). Individual δ^13^C_AAn_ values typically spanned between 0.4 to 0.6‰ compared to 2.6% and 5.2‰ for bulk δ^13^C values of *P. oceanica* and *M. pyrifera*, respectively.

**Figure 5:**
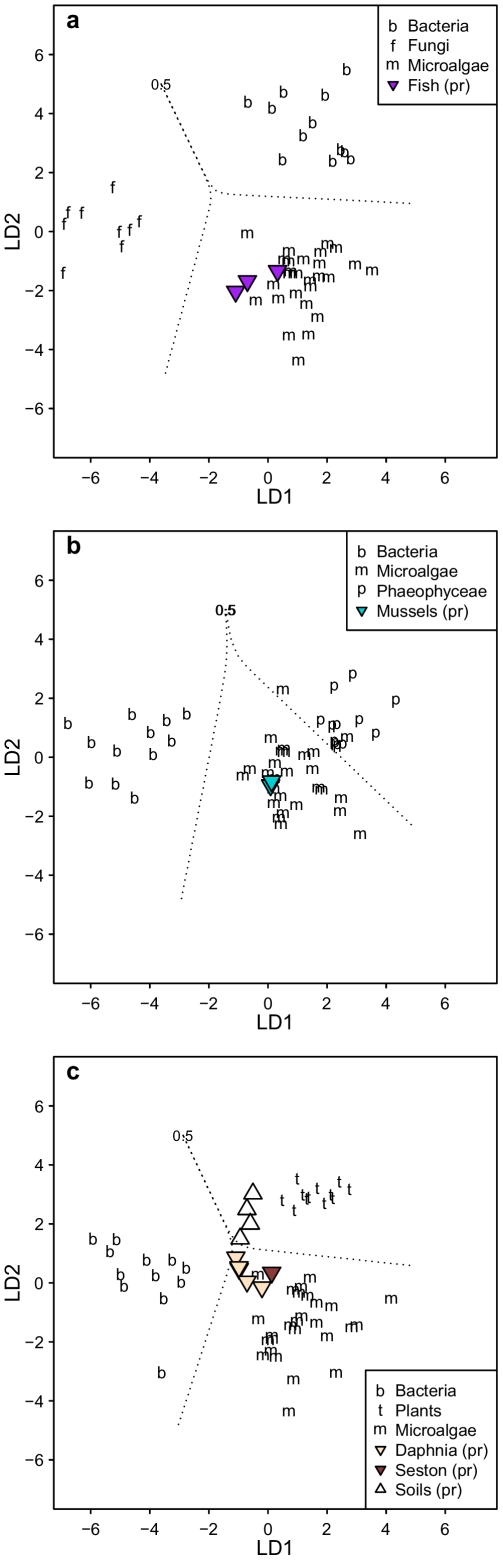
Application of source diagnostic δ^13^C_EAA_ patterns in food web studies across three different ecosystems. (a) In oligotrophic arctic lakes in Alaska, *Daphinia* sp. and seston cluster closely to each other, and their EAAs appear to derive predominantly from microalgae although a part of their EAAs may have come from foods reworked by bacteria or from allochtonous sources (i.e soils). (b) In the central North Pacific Ocean the EAAs of the carnivorous fish species (opah; *Lampris guttatus*, common dolphinfish; *Coryphaena hippurus*, broadbill swordfish; *Xiphias gladius*) resembled microalgae rather than EAAs from bacteria and fungi. (c) In a complex littoral marine system by the Californian shore, the δ^13^C_EAA_ fingerprints of California mussel (*Mytilus californianus*) resemble microalgae and not bacteria or brown algae, i.e. kelp. In the figure legend, ‘Pr’ signifies predicted samples. See Table S8 for analytical details.

**Figure 6:**
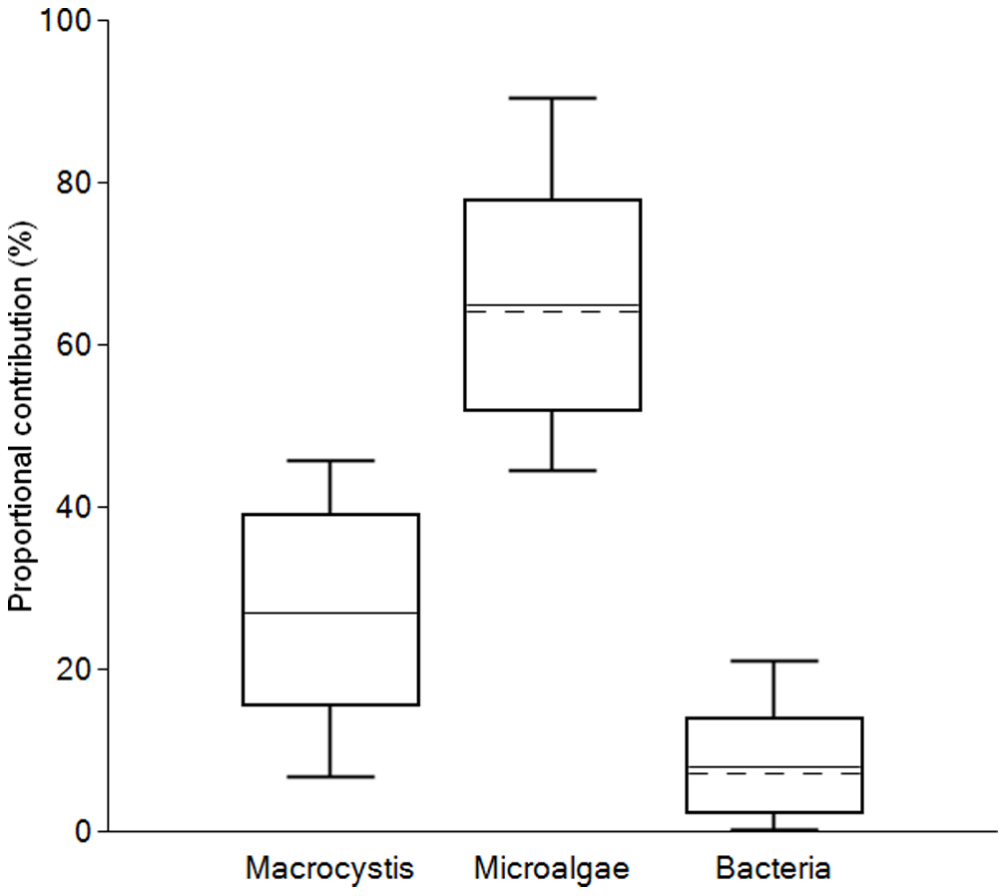
A boxplot generated with the FRUITS mixing model showing the contribution of essential amino acids (Leu, Lys, and Val) from three diets (bacteria; n = 12, kelp; n = 5, microalgae; n = 27) to the California mussel (*Mytilus californianus*; average value of two samples). The boxes provide a 68% confidence interval (corresponding to the 16^th^ and 84^th^ percentiles) and the whiskers provide a 95% confidence interval. The horizontal continuous line indicates the average while the horizontal discontinuous line indicates the median (50^th^ percentile). See Appendix S1 for detailed information.

Finally, we investigated how δ^13^C_EAA_ patterns of animals from three different aquatic ecosystems resembled the main primary production sources in their respective environments. For Arctic shallow lakes in Northern Alaska, we used bacteria, microalgae and terrestrial plants as the most likely end members for *Daphnia*. The LDA model classified all the categorical variables correctly, and the δ^13^C_EAA_ patterns of the five *Daphnia* samples resembled microalgae with 84.0±16.9% probability and bacteria with 11.9±12.3% probability (Fig. 5a, Table S8). The seston sample resembled microalgae with 96.8% probability. While three out of four soil samples resembled plants with >97% probability, the remaining sample resembled plants with 69% probability. In an open pelagic system, we used microalgae, bacteria and fungi as end members for three predatory fish species (*C. hippurus, L. guttatus and X. gladius*) from the central Pacific. The three categorical variables (algae, bacteria and fungi) were distinctly different and the δ^13^C_EAA_ patterns of all three fish samples matched those of microalgae with 100% probability (Fig. 5b, Table S8). In the estuarine system we selected microalgae, giant kelp and bacteria as the most likely particulate organic matter sources for the California mussel (*Mytilus californianus*). The LDA model classified all the categorical variables correctly, and the δ^13^C_EAA_ patterns of the two mussels resembled microalgae with ≥99.7% probability and brown algae with 0.3% probability (Fig. 5c, Table S8). We used the California mussel samples to exemplify how EAA isotope values can be used to obtain relative proportions of food sources for a consumer. The mixing model was based on δ^13^C_n_ values of the three most informative EAAs for separating microalgae, brown algae and bacteria: Leu, Val and Lys. For brown algae, we only considered kelp since it is the most dominant brown alga in the mussels’ habitat. We also included information about the relative proportion of the three EAAs (Leu, Lys and Val) in the food sources [27]–[29], and the likelihood that the mussels would consume one food sources over another (See Appendix S1). We found that the mussels obtained about two-third of their EAAs from microalgae (66.7±13.3%), about a quarter from kelp (26.0±11.4%) and the remaining fraction from bacteria (7.3±6.0%) (Fig. 6, Appendix S1).

## Discussion

Our results show that δ^13^C_AA_ patterns can be used as powerful and ubiquitous tracers for discerning carbon origins in both terrestrial and marine settings (Fig. 1). We found that δ^13^C values for seven out of eleven AAs were significantly different between algae and terrestrial plants, which enabled us to create a classification model that determined whether the AAs originated from terrestrial or marine sources (Figs. 2 and 3). The fact that variations in δ^13^C_AA_ patterns among algae were sufficiently constrained to make a clear distinction between aquatic and terrestrial primary producers seems remarkable, considering that our algal samples encompassed a large number of species from both freshwater and marine environments. Within the algal groups that comprised two macroalgal and five microalgal domains, brown algae stood out as having the most distinct δ^13^C_EAA_ patterns (Fig. 5c, Table S5). We also found that δ^13^C_EAA_ patterns of terrestrial and aquatic primary producers were different from bacteria and fungi. While the mechanistic reasons why algae vs. vascular plants have such unique δ^13^C_AA_ patterns are not currently clear as discussed below, the strong diagnostic potential we observe for these major classes of primary producers is consistent with broad ability of δ^13^C_AA_ patterns to distinguish between other major organism groups.

Our results also indicate that δ^13^C_EAA_ patterns applied as source diagnostic isotope fingerprints may offer a partial solution to one of the major issues for the application of stable isotopes in ecological and biogeochemical research: confounding variable source bulk isotope values. It has remained a persistent challenge for isotope ecologists to disentangle confounding δ^13^C values caused by variations in inorganic carbon sources and other environmental parameters. Here we show that despite substantial shifts in bulk δ^13^C values for the seagrass *P. oceanica* and the giant kelp *M. pyrifera* linked to season or growth conditions, δ^13^C_AAn_ values were constant within a 0.5‰ standard deviation (Fig. 4). These results are consistent with the notion that δ^13^C_AA_ patterns are mostly determined by major evolutionary AA metabolic pathways of an organism [11], [12], [30] rather than the factors affecting bulk δ^13^C values such as carbon availability, growth rates, and cell surface area [3]–[4]. For application in food web studies it is particularly encouraging that δ^13^C_AAn_ values only shifted by 0.5‰ compared to the >5‰ shift in bulk δ^13^C values of giant kelp. Another important observation is that light attenuation and leaf necrosis for the seagrass samples did not affect δ^13^C_AAn_ values notably. Light intensity and necrosis are important factors for seagrass growth and often associated with changes in leaf composition of phenolic compounds, carbohydrates and chlorophyll [31]–[33].

We directly tested the ability of δ^13^C_AA_ patterns to transfer information about major primary producer sources through food webs by examining δ^13^C_AA_ values in consumers from diverse habitats (freshwater arctic lakes, subtropical pelagic ocean, estuarine marine). In every habitat, the results were consistent with the hypothesis that δ^13^C_EAA_ source patterns based are conserved in passage through food webs. In the freshwater ecosystem, the resemblance of *Daphnia* and seston AAs with microalgae rather than bacteria and terrestrial plants agrees with limnological food web studies based on bulk isotope data [34] (Fig. 5a). However, it is interesting that neither *Daphnia* nor seston were projected directly on top of the algal samples pointing to a possible influence of microbial reworking or allochtonous input, as has been previously suggested in Arctic lakes [35]. In the open ocean almost all primary production derives from single-celled algae [36], so δ^13^C_AA_ patterns would be expected to align with algal sources in oceanic consumers. The δ^13^C_EAA_ patterns exactly followed this prediction (Fig. 5b), which also implies that δ^13^C_EAA_ diagnostic information was not substantially altered by microbial reworking [37]–[40], or transformed during digestive processes [41]. Our estuarine system is much more complex, with microalgae, giant kelp, and reworked organic matter as possible contributors to suspended particular organic matter (POM) [18]. For the estuarine classification of California mussels, we included bacteria in addition to microalgae and brown algae as end members in our model, and found that microalgae had a much higher probability being an AA source for the mussels than kelp (Fig. 5c). Since classification models are not suited to estimate relative proportions of food sources, we applied a mixing model to the study with estuarine mussels. Based on the three most informative EAAs for separating the mussel’s most likely food sources, we found that the mussels obtained about two-third of their EAAs from mussels, about a quarter from kelp and the remaining fraction from bacteria (Fig. 6, Appendix S1). Thus, our findings are consistent with the expectation that mussels mainly feed on POM derived from fresh phytoplankton, and that microbially reworked POM is a minor source [42].

If algae and vascular plants share similar amino acid biosynthetic pathways [43], [44], it raises the question why they have consistently different δ^13^C_AA_ patterns. It has been proposed by Hayes [30] that growth rates potentially could affect intramolecular isotope distribution. During the stationary growth phase the removal of carbon from the tricarboxylic acid cycle would be slower than in the exponential phase. This would lead to the accumulation of ^13^C enriched compounds at the ends of the biosynthetic pathways in turn delivering ^13^C depleted precursors to the first steps of the pathways [30]. While we did not test this hypothesis, the rather uniform ^13^C_AA_ patterns among microalgae with contrasting growth rates suggest that this influence is rather small. In support of this view, we found that the Mediterranean seagrass samples had similar δ^13^C_AA_ patterns in spite of the different lighting regimes and hence growth rates in their natural habitats. Isotope fractionation at metabolic branch points may also explain some of the observed differences in δ^13^C_AA_ patterns [11], [12,]. We found that algae were^ 13^C enriched relative to plants for the AAs belonging to the pyruvate group (Ala, Val, Leu). A similar case of greater ^13^C enrichment in algae than plants was found by Chikaraishi and Naraoka [44] for *n*-alkanes that like most other lipids have pyruvate as a precursor [45]. In contrast to the pyruvate AAs, the aromatic AAs (Tyr, Phe) were ^13^C depleted in algae relative to plants. From a carbon mass balance point of view it is possible that ^13^C enrichment of pyruvate AAs in algae lead to depletion of the aromatic AAs because they are less coupled to lipid synthesis by having phosphoenolpyruvate and erythrose-4-phosphate as precursors. The aromatic AAs also serve as precursors for the synthesis of numerous primary and secondary metabolites such as alkaloids and lignins [46], which also could have influenced ^13^C_AA_ fractionation of the aromatic AAs. For deepening our understanding of the biochemical processes leading to the ^13^C_AA_ fractionation patterns further inquiry is needed into the overall carbon mass balance between the most abundant hydrocarbon groups and AAs.

Taken together, our results show that δ^13^C_AA_ patterns can transcend bulk isotope analyses across diverse ecological environments, and be used to understand carbon sources and transfer in ecological research as exemplified with our classification and mixing modeling approaches. The strong diagnostic potential for algae and plants may be particularly powerful for applications in complex estuarine and coastal systems, where mixed aquatic and terrestrial inputs occur, or in freshwater environments with strong allochtonous influence. While our findings indicate that source-specificity of δ^13^C_EAA_ patterns is conserved across environmental gradients, further controlled physiological studies are also warranted to better understand under what circumstances these patterns may be altered. For a broad understanding of food web cycling of nutrients, we also stress that it is also important to consider other major biochemical classes, such as lipids and carbohydrates. Finally, in addition to investigating ecosystem level transfer of carbon and nitrogen, we suggest that this fingerprinting method can help assessing symbiotic contributions of AAs from bacteria to animal hosts. There is mounting evidence that protein-nitrogen assimilation is possible in the lower gut of some animals during digestion [47]–[49]. Our results indicate that δ^13^C_EAA_ patterns may offer a direct way to assess the importance of such microbial AA contributions, not only in the specific animals where this may occur, but more broadly up food chains.

## Supporting Information

Figure S1 **GC-C-IRMS chromatogram.**
(PDF)

Table S1 **Field sample characteristics.**
(PDF)

Table S2 **Laboratory sample characteristics.**
(PDF)

Table S3 **δ^13^C values of individual amino acids.**
(PDF)

Table S4 **Principal component analysis output for algal, bacterial, fungal and plant samples (** **Figure 1** **).**
(PDF)

Table S5 **ANOVA comparison of δ^13^C_AAn_ values.**
(PDF)

Table S6 **Linear discriminant analysis output for algal, bacterial, fungal and terrestrial plant samples (** **Figure 2** **).**
(PDF)

Table S7 **Linear discriminant function analysis output of aquatic primary producers (** **Figure 3** **).**
(PDF)

Table S8 **Linear discriminant function analysis output for amino acid origins of consumers (** **Figure 5** **).**
(PDF)

Appendix S1 **FRUITS mixing model data (** **Figure 6** **).**
(PDF)

## References

[1] Boecklen WJ, Yarnes CT, Cook BA, James AC (2011) On the Use of Stable Isotopes in Trophic Ecology. Annual Review of Ecology, Evolution, and Systematics 42: 411–440.

[2] Degens ET, Guillard RRL, Sackett WM, Hellebust JA (1968) Metabolic fractionation of carbon isotopes in marine plankton–I. Temperature and respiration experiments. Deep Sea Research and Oceanographic Abstracts 15: 1–9.

[3] Fogel ML, Cifuentes LA, Velinsky DJ, Sharp JH (1992) Relationship of Carbon Availability in Estuarine Phytoplankton to Isotopic Composition. Marine Ecology Progress Series 82: 291–300.

[4] Popp BN, Laws EA, Bidigare RR, Dore JE, Hanson KL, et al (1998) Effect of phytoplankton cell geometry on carbon isotopic fractionation. Geochimica et Cosmochimica Acta 62: 69–77.

[5] Jones WB, Cifuentes LA, Kaldy JE (2003) Stable carbon isotope evidence for coupling between sedimentary bacteria and seagrasses in a sub-tropical lagoon. Marine Ecology-Progress Series 255: 15–25.

[6] De Troch M, Boeckx P, Cnudde C, Van Gansbeke D, Vanreusel A, et al (2012) Bioconversion of fatty acids at the basis of marine food webs: insights from a compound-specific stable isotope analysis. Marine Ecology Progress Series 465: 53–67.

[7] Hedges JI, Baldock JA, Gelinas Y, Lee C, Peterson M, et al (2001) Evidence for non-selective preservation of organic matter in sinking marine particles. Nature 409: 801–804. 11236989 10.1038/35057247

[8] McMahon KW, Fogel ML, Elsdon TS, Thorrold SR (2010) Carbon isotope fractionation of amino acids in fish muscle reflects biosynthesis and isotopic routing from dietary protein. Journal of Animal Ecology 79: 1132–1141. 20629794 10.1111/j.1365-2656.2010.01722.x

[9] O’Brien DM, Fogel ML, Boggs CL (2002) Renewable and nonrenewable resources: Amino acid turnover and allocation to reproduction in lepidoptera. Proceedings of the National Academy of Sciences of the United States of America 99: 4413–4418. 11930002 10.1073/pnas.072346699 PMC123662

[10] Scott JH, O’Brien DM, Emerson D, Sun H, McDonald GD, et al (2006) An examination of the carbon isotope effects associated with amino acid biosynthesis. Astrobiology 6: 867–880. 17155886 10.1089/ast.2006.6.867

[11] Larsen T, Taylor D, Leigh MB, O’Brien DM (2009) Stable isotope fingerprinting: a novel method for identifying plant, fungal or bacterial origins of amino acids. Ecology 90: 3526–3535. 20120819 10.1890/08-1695.1

[12] Hayes JM (2001) Fractionation of the Isotopes of Carbon and Hydrogen in Biosynthetic Processes. In: Cole JWVaDR, editor. Reviews in Mineralogy and Geochemistry 43, Stable Isotope Geochemistry. Washington The Mineralogical Society of America. 225–277.

[13] Larsen T, Ventura M, O’Brien DM, Magid J, Lomstein BA, et al (2011) Contrasting effects of nitrogen limitation and amino acid imbalance on carbon and nitrogen turnover in three species of Collembola. Soil Biology and Biochemistry 43: 749–759.

[14] Larsen T, Wooller MJ, Fogel ML, O’Brien DM (2012) Can amino acid carbon isotope ratios distinguish primary producers in a mangrove ecosystem? Rapid Communications in Mass Spectrometry 26: 1541–1548. 22638971 10.1002/rcm.6259

[15] Goericke G, Montoya JP, Fry B (1994) Physiology of isotopic fractionation in algae and cyanobacteria. In: Lajtha K, Michener RH, editors. Stable isotopes in ecology and environmental science. Oxford; Boston: Blackwell Scientific Publications. 187–221.

[16] Cloern JE, Canuel EA, Harris D (2002) Stable carbon and nitrogen isotope composition of aquatic and terrestrial plants of the San Francisco Bay estuarine system. Limnology and Oceanography 47: 713–729.

[17] Bouillon S, Connolly RM, Lee SY (2008) Organic matter exchange and cycling in mangrove ecosystems: Recent insights from stable isotope studies. Journal of Sea Research 59: 44–58.

[18] Foley MM, Koch PL (2010) Correlation between allochthonous subsidy input and isotopic variability in the giant kelp *Macrocystis pyrifera* in central California, USA. Marine Ecology Progress Series 409: 41–50.

[19] Choy CA, Popp BN, Kaneko JJ, Drazen JC (2009) The influence of depth on mercury levels in pelagic fishes and their prey. Proceedings of the National Academy of Sciences of the United States of America 106: 13865–13869. 19666614 10.1073/pnas.0900711106 PMC2728986

[20] Amelung W, Zhang X (2001) Determination of amino acid enantiomers in soils. Soil Biology & Biochemistry 33: 553–562.

[21] He HB, Lu HJ, Zhang W, Hou SM, Zhang XD (2011) A liquid chromatographic/mass spectrometric method to evaluate ^13^C and ^15^N incorporation into soil amino acids. Journal of Soils and Sediments 11: 731–740.

[22] Corr LT, Berstan R, Evershed RP (2007) Development of N-acetyl methyl ester derivatives for the determination of δ^13^C values of amino acids using gas chromatography-combustion-isotope ratio mass spectrometry. Analytical Chemistry 79: 9082–9090. 17973497 10.1021/ac071223b

[23] Silfer JA, Engel MH, Macko SA, Jumeau EJ (1991) Stable carbon isotope analysis of amino acid enantiomers by conventional isotope ratio mass spectrometry and combined gas chromatography/isotope ratio mass spectrometry. Anal Chem 63: 370–374.

[24] R-Development-Core-Team (2012) R: A language and environment for statistical computing. Vienna, Austria: R Foundation for Statistical Computing.

[25] Venables WN, Ripley BD (2002) Modern applied statistics with S. New York: Springer. xi, 495 p.

[26] Fernandes R, Rinne C, Nadeau M-J, Grootes PM (2012) Revisiting the chronology of northern German monumentality sites: preliminary results. In: Hinz M, Müller J, Lüth F (eds.). Frühe Monumentalität und soziale Differenzierung, Band 2, Verlag Dr. Rudolf Habelt GmbH.

[27] Mateus H, Regenstein JM, Baker RC (1976) The amino acid composition of the marine brown alga *Macrocystis pyrifera* from Baja California, Mexico. Botanica Marina. 155–156.

[28] Brown MR (1991) The amino-acid and sugar composition of 16 species of microalgae used in mariculture. Journal of Experimental Marine Biology and Ecology 145: 79–99.

[29] Simon M, Azam F (1989) Protein content and protein synthesis rates of planktonic marine bacteria. Marine Ecology Progress Series 51: 201–213.

[30] Hayes JM (1993) Factors controlling ^13^C contents of sedimentary organic compounds: Principles and evidence. Marine Geology 113: 111–125.

[31] Dennison WC, Alberte RS (1982) Photosynthetic Responses of *Zostera marina* L. (Eelgrass) to in Situ Manipulations of Light Intensity. Oecologia 55: 137–144. 28311224 10.1007/BF00384478

[32] Zimmerman RC, Kohrs DG, Steller DL, Alberte RS (1995) Carbon Partitioning in Eelgrass - Regulation by Photosynthesis and the Response to Daily Light-Dark Cycles. Plant Physiology 108: 1665–1671. 12228571 10.1104/pp.108.4.1665 PMC157548

[33] Agostini S, Desjobert J-M, Pergent G (1998) Distribution of phenolic compounds in the seagrass *Posidonia oceanica* . Phytochemistry 48: 611–617. 10.1016/j.phytochem.2004.09.003 15643707

[34] Francis TB, Schindler DE, Holtgrieve GW, Larson ER, Scheuerell MD, et al (2011) Habitat structure determines resource use by zooplankton in temperate lakes. Ecology Letters 14: 364–372. 21314881 10.1111/j.1461-0248.2011.01597.x

[35] Rautio M, Vincent WF (2007) Isotopic analysis of the sources of organic carbon for zooplankton in shallow subarctic and arctic waters. Ecography 30: 77–87.

[36] Falkowski PG (1980) Primary productivity in the sea. New York: Plenum Press. ix, 531 p.

[37] Keil RG, Fogel ML (2001) Reworking of amino acid in marine sediments: Stable carbon isotopic composition of amino acids in sediments along the Washington coast. Limnology and Oceanography 46: 14–23.

[38] Macko SA, Estep MLF (1984) Microbial alteration of stable nitrogen and carbon isotopic compositions of organic matter. Organic Geochemistry 6: 787–790.

[39] Kaiser K, Benner R (2008) Major bacterial contribution to the ocean reservoir of detrital organic carbon and nitrogen. Limnology and Oceanography 53: 99–112.

[40] McCarthy MD, Benner R, Lee C, Hedges JI, Fogel ML (2004) Amino acid carbon isotopic fractionation patterns in oceanic dissolved organic matter: an unaltered photoautotrophic source for dissolved organic nitrogen in the ocean? Marine Chemistry 92: 123–134.

[41] Newsome SD, Fogel ML, Kelly L, del Rio CM (2011) Contributions of direct incorporation from diet and microbial amino acids to protein synthesis in *Nile tilapia* . Functional Ecology 25: 1051–1062.

[42] Miller RJ, Page HM (2012) Kelp as a trophic resource for marine suspension feeders: a review of isotope-based evidence. Marine Biology 159: 1391–1402.

[43] Falkowski PG, Katz ME, Knoll AH, Quigg A, Raven JA, et al (2004) The Evolution of Modern Eukaryotic Phytoplankton. Science 305: 354–360. 15256663 10.1126/science.1095964

[44] Chikaraishi Y, Naraoka H (2003) Compound-specific δ^13^C analyses of n-alkanes extracted from terrestrial and aquatic plants. Phytochemistry 63: 361–371. 12737985 10.1016/s0031-9422(02)00749-5

[45] Zhou YP, Grice K, Stuart-Williams H, Farquhar GD, Hocart CH, et al (2010) Biosynthetic origin of the saw-toothed profile in δ^13^C and δ^2^H of *n*-alkanes and systematic isotopic differences between *n-, iso- and anteiso*-alkanes in leaf waxes of land plants. Phytochemistry 71: 388–403. 20056262 10.1016/j.phytochem.2009.11.009

[46] Buchanan BB, Gruissem W, Jones RL (2000) Biochemistry & molecular biology of plants. Rockville, Md.: American Society of Plant Physiologists. xxxix, 1367 p.

[47] Binder HJ (1970) Amino acid absorption in the mammalian colon. Biochimica et Biophysica Acta 219: 503–506. 5497209 10.1016/0005-2736(70)90233-6

[48] Ugawa S, Sunouchi Y, Ueda T, Takahashi E, Saishin Y, et al (2001) Characterization of a mouse colonic system B(0+) amino acid transporter related to amino acid absorption in colon. American Journal of Physiology - Gastrointestinal and Liver Physiology 281: G365–G370. 11447016 10.1152/ajpgi.2001.281.2.G365

[49] Ford D, Howard A, Hirst BH (2003) Expression of the peptide transporter hPepT1 in human colon: a potential route for colonic protein nitrogen and drug absorption. Histochemistry and Cell Biology 119: 37–43. 12548404 10.1007/s00418-002-0479-y

