# Correction: Tracing Carbon Sources through Aquatic and Terrestrial Food Webs Using Amino Acid Stable Isotope Fingerprinting

**DOI:** 10.1371/annotation/2f7a422e-13a9-4491-ae81-4fd215149654

**Published:** 2014-01-06

**Authors:** Thomas Larsen, Marc Ventura, Nils Andersen, Diane M. O’Brien, Uwe Piatkowski, Matthew D. McCarthy

There is an error in Figure 5. Please view the correct figure here: http://plosone.org/corrections/pone.0073441.g005.cn.tif

There is also an error in the second to last sentence of the third paragraph in the "Discussion" section. The correct sentence is:

"Based on the three most informative EAAs for separating the mussel’s most likely food sources, we found that the mussels obtained about two-third of their EAAs from microalgae, about a quarter from kelp and the remaining fraction from bacteria (Fig. 6, Appendix S1)."

