# Supplementary material for: Tracing Carbon Sources through Aquatic and Terrestrial Food Webs Using Amino Acid Stable Isotope Fingerprinting
Source: PLoS One. 2013 Sep 17;8(9):e73441. doi: 10.1371/journal.pone.0073441 (PMC3775739; doi:10.1371/journal.pone.0073441)

Supporting Figure S1

GC-C-IRMS chromatogram of the diatom *Amphora coffaeiformis* with the ratio of 45 to 44 voltages over time in the upper panel and voltage (mV) over time of ion masses 44 and 45, the most abundant CO_2 ion forms, in the lower panel.

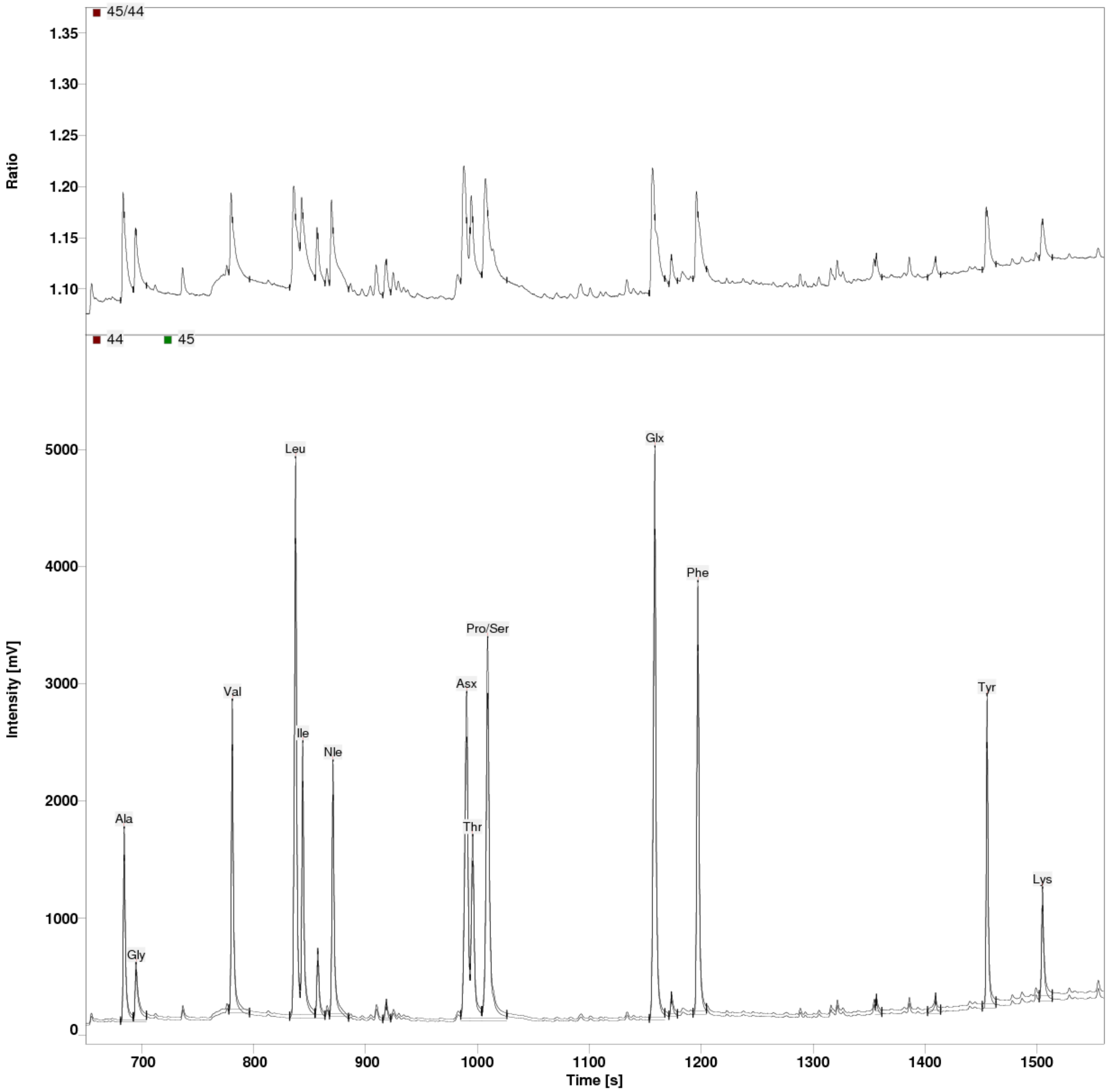

Supplement: Figure S1 — GC-C-IRMS chromatogram. (PDF) [file pone.0073441.s001.pdf]
