# Supplementary material for: Tracing Carbon Sources through Aquatic and Terrestrial Food Webs Using Amino Acid Stable Isotope Fingerprinting
Source: PLoS One. 2013 Sep 17;8(9):e73441. doi: 10.1371/journal.pone.0073441 (PMC3775739; doi:10.1371/journal.pone.0073441)
Supplement: Table S1 — Field sample characteristics. (PDF) [file pone.0073441.s002.pdf]

Supporting Table S1

Identities, sampling locations, elemental C and N content and bulk isotope values of field samples.

Species	Code	Phylogeny	Location	Date	Latitude	Longitude	%C	%N	C:N (atomic)	Bulk $\delta^{13}C$	Bulk $\delta^{15}N$
Terestrial plants											
Quercus robur	T1	Fagaceae	Silkeborg, DK	Jul-09	56.12°	9.62°	46.0	2.2	17.8	-30.9	-5.8
Alnus glutinosa	T2	Betulaceae	Silkeborg, DK	Jul-09	56.12°	9.62°	36.1	2.3	13.2	-31.7	-5.3
Salix sp	T3	Salicaceae	North Slope, AK	Jul-08	70.10°	-148.52°	42.4	2.7	13.5	-29.3	0.2
Polygonum viviparum	T4	Polygonaceae	North Slope, AK	Jul-08	70.10°	-148.52°	45.0	2.5	15.3	-29.1	1.9
Carex aquatilis	T5	Cyperaceae	North Slope, AK	Jul-08	70.05°	-148.60°	44.3	2.0	19.5	-27.2	4.9
Calamagrostis canadensis	T6	Poaceae	North Slope, AK	Jul-08	70.05°	-148.60°	46.5	2.5	15.9	-27.3	5.6
Menyanthes trifoliata	T7	Menyanthaceae	North Slope, AK	Jul-08	68.80°	-148.84°	44.0	3.0	12.8	-28.2	5.6
Betula nana	T8	Betulaceae	North Slope, AK	Jul-08	68.80°	-148.84°	48.8	2.3	17.8	-28.2	-2.3
Carex utriculata	T9	Cyperaceae	North Slope, AK	Jul-08	68.68°	-149.08°	44.2	1.4	26.5	-27.2	2.3
Salix reticulata	T10	Malpighiales	North Slope, AK	Jul-08	68.68°	-149.08°	46.7	2.9	13.8	-26.2	-0.1
Eriophorum angustifolium	T11	Cyperaceae	North Slope, AK	Jul-08	68.65°	-149.61°	59.4	2.5	20.2	-26.6	2.1
Rumex arcticus	T12	Polygonaceae	North Slope, AK	Jul-08	68.65°	-149.61°	44.3	2.2	17.0	-27.2	2.3
Seagrasses											
Posidonia oceanica	S1	Posidoniaceae	Medes, ES	Sep/Oct-08	42.05°	3.22°	45.5	1.8	22.3	-11.1	2.6
Posidonia oceanica	S2	Posidoniaceae	Fenals, ES	Sep/Oct-08	41.69°	2.84°	45.4	1.5	25.5	-11.7	2.0
Posidonia oceanica	S3	Posidoniaceae	Sitges, ES	Sep/Oct-08	41.22°	1.78°	46.3	1.7	23.3	-12.7	4.4
Posidonia oceanica	S4	Posidoniaceae	Salou, ES	Sep/Oct-08	41.06°	1.17°	43.0	2.0	18.6	-13.7	5.0
Posidonia oceanica	S5	Posidoniaceae	Ametlla, ES	Sep/Oct-08	40.87°	0.80°	44.6	1.6	23.7	-13.8	4.2
Phyllospadix scouleri	S6	Zosteraceae	Santa Cruz, CA	Spring-09	36.95º	-122.06º	40.6	3.1	11.2	-14.7	8.9
Phyllospadix torreyi	S7	Zosteraceae	Santa Cruz, CA	Spring-09	36.95º	-122.06º	37.7	2.8	11.7	-15.9	7.9
Macroalgae											
Prionitis sp	R1	Rhodophyta	Santa Cruz, CA	Mar-09	36.95º	-122.06º	34.7	5.0	5.9	-11.8	9.9
Osmundea spectabilis	R2	Rhodophyta	Santa Cruz, CA	Mar-09	36.95º	-122.06º	28.6	3.1	7.8	-15.1	9.7
Chondracanthus canaliculatus	R3	Rhodophyta	Santa Cruz, CA	Mar-09	36.95º	-122.06º	31.8	2.8	9.7	-19.2	8.5
Calliarthron sp	R4	Rhodophyta	Santa Cruz, CA	Mar-09	36.95º	-122.06º	4.8	0.6	7.4	-15.1	5.5
Corallina sp	R5	Rhodophyta	Santa Cruz, CA	Mar-09	36.95º	-122.06º	7.5	0.8	7.9	-15.8	7.6
Odonthalia floccosa	R6	Rhodophyta	Santa Cruz, CA	Mar-09	36.95º	-122.06º	35.8	5.1	6.0	-15.6	9.8
Mastocarpus sp.	R7	Rhodophyta	Santa Cruz, CA	Mar-09	36.95º	-122.06º	61.3	6.1	8.6	-15.2	9.9
Endocladia muricata	R8	Rhodophyta	Santa Cruz, CA	Mar-09	36.95º	-122.06º	39.3	3.7	9.1	-16.8	9.6
Mazzaella flaccida	R9	Rhodophyta	Santa Cruz, CA	Mar-09	36.95º	-122.06º	30.1	2.8	9.3	-16.4	9.8
Macrocystis pyrifera	P1	Phaeophyceae	Big Sur, CA	Nov-06	36.28º	-121.87º	32.0	1.9	14.6	-14.9	9.2
Macrocystis pyrifera	P2	Phaeophyceae	Big Sur, CA	Aug-05	36.28º	-121.86º	34.6	2.4	12.6	-14.3	9.4
Macrocystis pyrifera	P3	Phaeophyceae	Big Sur, CA	May-06	36.28º	-121.86º	36.9	2.2	14.3	-17.8	8.4
Macrocystis pyrifera	P4	Phaeophyceae	Big Sur, CA	Sep-06	36.28º	-121.86º	32.1	2.2	12.5	-13.4	9.7

Continues on next page

Table S1 continued

	Species	Code	Phylogeny	Location	Date	Latitude	Longitude	%C	%N	C:N (atomic)	Bulk $\delta^{13}C$	Bulk $\delta^{15}N$
	Macrocystis pyrifera	P5	Phaeophyceae	Big Sur, CA	Mar-07	36.28º	-121.86º	33.3	1.8	16.1	-18.7	7.2
	Scytosiphon sp	P6	Phaeophyceae	Santa Cruz, CA	Mar-09	36.95º	-122.06º	32.9	2.9	9.8	-8.5	8.7
	Laminaria sp	P7	Phaeophyceae	Santa Cruz, CA	Mar-09	36.95º	-122.06º	25.2	1.8	12.0	-14.3	9.9
	Silvetia sp	P8	Phaeophyceae	Santa Cruz, CA	Mar-09	36.95º	-122.06º	37.5	1.5	21.3	-15.2	9.3
	Petrospongium sp	P9	Phaeophyceae	Santa Cruz, CA	Mar-09	36.95º	-122.06º	30.8	1.9	13.7	-11.9	9.6
	Pelvetiopsis sp.	P10	Phaeophyceae	Santa Cruz, CA	Mar-09	36.95º	-122.06º	32.2	1.8	15.2	-17.9	10.0
	Ralfsia sp	P11	Phaeophyceae	Santa Cruz, CA	Mar-09	36.95º	-122.06º	34.8	2.2	13.5	-7.0	9.6
	Cystoseira osmundacea	P12	Phaeophyceae	Santa Cruz, CA	Mar-09	36.95º	-122.06º	28.6	1.8	13.8	-17.0	8.8
Fish												
	Coryphaena hippurus	ch	Coryphaenidae	CN Pacific Ocean	Jun-10	~25º	~-150º	NA	NA	NA	-16.3	NA
	Lampris guttatus	lg	Lampridae	CN Pacific Ocean	Dec-10	~22º	~-140º	NA	NA	NA	-19.9	NA
	Xiphias gladius	xg	Xiphiidae	CN Pacific Ocean	Jan-11	~31º	~-135º	NA	NA	NA	-21.8	NA
Mussels												
	Mytilus californianus	gav	Mytilidae	Gaviota, CA	Jan-11	34.47º	-120.48º	NA	NA	NA	-15.0	10.3
	Mytilus californianus	sc	Mytilidae	Santa Cruz, CA	Jan-11	36.95º	-122.05º	NA	NA	NA	-13.3	10.8
Daphnia												
	Daphnia middendorffiana	dL1	Daphnia	North Slope, AK	Jul-08	70.10°	-148.52°	49.0±0.4	11.5±0.3	3.6±0.0	-26.9±0.0	NA
	Daphnia middendorffiana	dL2	Daphnia	North Slope, AK	Jul-08	70.05°	-148.60°	49.0±0.3	11.5±0.1	3.7±0.1	-26.6±0.0	3.4±0.3
	Daphnia tenebrosa	dL3	Daphnia	North Slope, AK	Jul-08	68.80°	-148.84°	50.3±3.8	11.0±1.0	3.9±0.3	-26.3±0.3	1.3±0.0
	Daphnia pulex	dL4	Daphnia	North Slope, AK	Jul-08	68.68°	-149.08°	48.6±1.4	11.6±0.3	3.6±0.0	-25.5±0.1	1.8±0.5
	Daphnia pulex	dL5	Daphnia	North Slope, AK	Jul-08	68.65°	-149.61°	49.9±1.8	12.1±0.5	3.5±0.0	-25.7±0.1	2.5±1.0
Soils												
	Peat soil	sL1	Detrital	North Slope, AK	Jul-08	70.10°	-148.52°	22	0.9	21.4	-27.7	2.9
	Peat soil	sL3	Detrital	North Slope, AK	Jul-08	68.80°	-148.84°	32.2	1.2	22.8	-26.8	3.4
	Peat soil	sL4	Detrital	North Slope, AK	Jul-08	68.68°	-149.08°	39.2	2	16.5	-26.3	0.7
	Peat soil	sL5	Detrital	North Slope, AK	Jul-08	68.65°	-149.61°	38.1	1.7	19.2	-25.7	1
Seston												
	Seston 5 µm	pL1	Plankton	North Slope, AK	Jul-08	70.10°	-148.52°	15.5	1.1	11.9	-20.1	1.7
	Seston 5 µm	pL2	Plankton	North Slope, AK	Jul-08	70.05°	-148.60°	35.6	4	7.6	-33.6	0.1
