# Supplementary material for: Tracing Carbon Sources through Aquatic and Terrestrial Food Webs Using Amino Acid Stable Isotope Fingerprinting
Source: PLoS One. 2013 Sep 17;8(9):e73441. doi: 10.1371/journal.pone.0073441 (PMC3775739; doi:10.1371/journal.pone.0073441)
Supplement: Table S2 — Laboratory sample characteristics. (PDF) [file pone.0073441.s003.pdf]

Supporting Table S2

Identities, origins, substrates, elemental C and N content and bulk isotope values of laboratory samples.

Phylogeny	Code	Origin	Nutrient source[$]	Sub-strate	ºC	%C	%N	C:N (atomic)	Bulk $\delta^{13}C$	Bulk $\delta^{15}N$
Bacteria										
Burkholderia xenovorans	B1	culture collection*	MAG [1,2]	Agar	22	47.2	12.5	3.2	-12.5	-8.1
Methylobacterium sp	B2	isolated from boreal forest*	MAG	Liquid	25	44.8	9.0	4.3	-13.3	-10.0
Klebsiella sp	B3	isolated from boreal forest*	MAG	Agar	22	45.1	11.0	3.5	-22.9	-4.0
Rhodococcus spp.	B4	isolated from boreal forest*	Czapek	Liquid	25	54.9	4.9	9.5	-22.7	5.1
Unidentified	B5	isolated from tundra soil	MAG	Agar	22	44.2	11.2	3.4	-16.2	-0.1
Unidentified	B6	isolated from tundra soil	MAG	Agar	22	43.6	10.6	3.5	-16.6	2.1
Unidentified	B7	isolated from tundra soil	MAG	Agar	22	44.1	10.4	3.6	-16.2	0.4
Unidentified	B8	isolated from arctic lake	MAG	Agar	22	44.2	10.4	3.7	-15.5	0.1
Unidentified	B9	isolated from tundra soil	MAG	Agar	22	43.7	10.3	3.6	-22.9	-7.0
Unidentified	B10	isolated from tundra soil	MAG	Agar	22	40.3	7.9	4.4	-17.4	-1.1
Unidentified	B11	isolated from tundra soil	MAG	Agar	22	41.7	8.4	4.3	-17.9	0.9
Unidentified	B12	isolated from arctic lake	MAG	Agar	22	42.5	9.9	3.7	-23.2	-2.5
Fungi										
Ascomycota	F1	isolated from boreal forest*	Czapek	Liquid	25	47.3	1.9	21.1	-25.0	2.8
Aureobasidium pullulans	F2	isolated from boreal forest*	MAG	Liquid	25	41.5	6.0	5.9	-22.6	2.6
Bionectria orhroleuca	F3	isolated from boreal forest*	Czapek	Agar	22	42.5	4.5	8.2	-24.6	4.0
Nectria vilior	F4	isolated from boreal forest*	Czapek	Liquid	25	34.7	2.9	10.3	-23.6	2.8
Mortierella alpina	F8	culture collection*	MMN [3]	Agar	22	40.5	6.0	5.8	-8.7	9.5
Unidentified	F5	isolated from tundra soil	MMN	Liquid	25	53.6	2.3	19.8	-10.7	6.5
Unidentified	F6	isolated from tundra soil	MMN	Liquid	25	55.0	1.6	29.4	-11.3	8.7
Unidentified	F7	isolated from tundra soil	MMN	Liquid	25	47.9	4.0	10.3	-10.9	8.4
Unidentified	F9	isolated from tundra soil	MMN	Liquid	25	49.9	3.3	12.9	-10.9	8.2
Microalgae										
Cyanothece sp	C1	GEOMAR[†]	no nutrients added	SW	29	42.9	3.7	9.9	-20.1	-1.2
Merismopedia punctata	C2	GEOMAR	f/2 diluted 1:4 [4,5]	BW	20	20.1	3.1	5.6	-25.9	5.4
Anabaena cylindrica	C3	SAG[‡]	no nutrients added	FW	20	46.6	9.0	4.5	-19.3	-1.0
Nostoc muscorum	C4	SAG	no nutrients added	FW	20	50.5	6.3	6.9	-19.1	3.6
Achnanthes brevipes	D1	GEOMAR	f/2	BW	20	28.8	1.9	13.1	-12.8	4.6
Amphora coffaeiformis	D2	GEOMAR	f/2	BW	20	24.7	2.2	9.6	-10.5	5.5
Melosira varians	D3	GEOMAR	f/2	BW	20	24.0	3.2	6.5	-14.6	4.0
Phaeodactylum tricornutum	D4	SAG	f/2	SW	20	18.8	3.1	5.1	-19.4	10.4
Stauroneis constricta	D5	GEOMAR	f/2	SW	20	24.7	1.2	17.6	-9.1	5.1

Continues on next page

Table S2 continued

Phylogeny	Code	Origin	Nutrient source$	Sub-strate	ºC	%C	%N	C:N (atomic)	Bulk $\delta^{13}C$	Bulk $\delta^{15}N$
Microalgae										
Emiliana huxleyi	H1	GEOMAR	f/2 w/o silica	SW	20	NA	NA	NA	NA	NA
Emiliana huxleyi	H2	GEOMAR	f/2 w/o silica	BW	20	NA	NA	NA	NA	NA
Isochrysis galbana	H3	GEOMAR	f/2 w/o silica	BW	20	NA	NA	NA	NA	NA
Corcontochrysis noctivaga	H4	SAG	no nutrients added	FW	20	NA	NA	NA	NA	NA
Dunaliella sp.	K1	GEOMAR	f/2 w/o silica	SW	20	10.3	1.6	5.5	-16.3	8.9
Prasinocladus marinus	K2	GEOMAR	f/2 w/o silica	BW	20	38.4	4.0	8.3	-17.0	8.4
Ankistrodesmus falcatus	K3	SAG	f/2 w/o silica	FW	20	NA	NA	NA	NA	NA
Chlamydocapsa maxima	K4	SAG	no nutrients added	FW	20	NA	NA	NA	NA	NA
Chlamydomonas asymmetrica	K5	SAG	f/2 w/o silica	FW	20	48.6	2.8	14.8	-19.3	1.9
Chlamydomonas gigantea	K6	SAG	f/2 w/o silica	FW	20	NA	NA	NA	NA	NA
Ochromonas minima	X1	GEOMAR	JM SW modified[6]	BW	20	29.2	4.4	5.7	-27.7	-8.8
Ochromonas villosa	X2	GEOMAR	JM SW modified	BW	20	40.6	5.9	5.9	-25.7	-3.4
Ochromonas danica	X3	SAG	no nutrients added	FW	20	40.5	7.0	5.0	-21.4	-3.5
Chromulina sp	X4	SAG	no nutrients added	FW	20	NA	NA	NA	NA	NA
Cryptomonas sp	Y1	SAG	no nutrients added	FW	20	45.7	4.3	9.1	-15.6	3.9
Composite natural sample§	N1	Kiel fjord	no nutrients added	BW	16	NA	NA	NA	NA	NA
Composite natural sample§	N2	Kiel fjord	no nutrients added	BW	16	NA	NA	NA	NA	NA
Composite natural sample§	N3	Kiel fjord	no nutrients added	BW	16	NA	NA	NA	NA	NA

Table footnotes.*Samples were obtained from the study Larsen et al. [7]; $ Carbon and nitrogen sources in bacterial and fungal media: Czapek contains 30 g/L sucrose and 3 g/L sodium nitrate, modified arabinose gluconate (MAG) contains 1 g/L L-arabinose, 1 g/L D-gluconic acid, and 0.32 g/L ammonium chloride and Melin-Norkrans (MMN) agar medium contains 10 g/L D-glucose and 0.25 g/L ammonium phosphate. See Larsen et al. [7] for $\delta^{13}C$ values of the substrates. Algae were grown at 12h/12h dark/light cycles at 80-100 (uE/m^{2}s) in either natural brackish (BW) or seawater (SW). † GEOMAR - Helmholtz Centre for Ocean Research Kiel; ‡ SAG - The Culture Collection of Algae at Goettingen University. §We estimated by visual inspection that diatoms comprised between 60 and 80% of the algal cells in the natural samples.

S2 references

1. Cole MA, Elkan GH (1973) Transmissible Resistance to Penicillin-G, Neomycin, and Chloramphenicol in *Rhizobium japonicum*. Antimicrobial Agents and Chemotherapy 4: 248-253.
2. Berkum Pv (1990) Evidence for a Third Uptake Hydrogenase Phenotype among the Soybean Bradyrhizobia. Appl Environ Microbiol 56: 3835–3841.
3. Marx A, Striegel K, deGraaf AA, Sahm H, Eggeling L (1997) Response of the central metabolism of Corynebacterium glutamicum to different flux burdens. Biotechnology and Bioengineering 56: 168-180.
4. Guillard RRL (1975) Culture of phytoplankton for feeding marine invertebrates. In: Smith WL, Chanley MH, editors. Culture of marine invertebrate animals. New York: Plenum Book Publ. Corp.
5. Provasoli-Guillard National Center for Marine Algae and Microbiota. f/2 Medium, < https://ncma.bigelow.org/> (2012).
6. Roscoff Culture Collection. JM SW modified, <http://www.sb-roscoff.fr/Phyto/RCC > (2012).
7. Larsen T, Taylor D, Leigh MB, O'Brien DM (2009) Stable isotope fingerprinting: a novel method for identifying plant, fungal or bacterial origins of amino acids. Ecology 90: 3526-3535.
