# Supplementary material for: Tracing Carbon Sources through Aquatic and Terrestrial Food Webs Using Amino Acid Stable Isotope Fingerprinting
Source: PLoS One. 2013 Sep 17;8(9):e73441. doi: 10.1371/journal.pone.0073441 (PMC3775739; doi:10.1371/journal.pone.0073441)
Supplement: Table S3 — δ13C values of individual amino acids. (PDF) [file pone.0073441.s004.pdf]

Supporting Table S3

Amino acid $\delta^{13}C$ values (‰). Each sample was analyzed in triplicate (mean±stdev). See Table S1 and S2 for sample identities. NA indicates missing values.

ID	Ala	Asx	Glx	Gly	His	Ile	Leu	Lys	Met	Phe	Thr	Tyr	Val
Bacteria													
B1	-11.3± 0.1	-12.9± 0.9	-11.5± 0.0	-12.9± 0.1	-8.4± 0.9	-12.0± 0.0	-12.8± 0.1	-10.4± 0.1	-18.6± 0.1	-18.8± 0.0	-5.2± 0.8	-16.8± 0.1	-13.6± 0.1
B2	-12.2± 0.0	-17.6± 0.3	-17.8± 0.0	-13.9± 0.0	-8.1± 0.7	-12.9± 0.1	-13.0± 0.0	-14.9± 0.1	-18.5± 0.2	-17.0± 0.1	-8.2± 1.4	-18.1± 0.1	-14.1± 0.1
B3	-15.9± 0.0	-21.8± 0.7	-18.3± 0.0	-21.3± 0.2	-16.0± 0.4	-18.9± 0.2	-19.9± 0.0	-20.3± 0.2	-25.7± 0.3	-24.6± 0.1	-15.0± 0.8	-22.9± 0.0	-20.8± 0.0
B4	-22.6± 0.0	-22.7± 0.4	-22.9± 0.0	-25.3± 0.1	-22.2± 0.4	-23.7± 0.1	-25.2± 0.2	-21.2± 1.1	-27.5± 0.2	-28.9± 0.1	-15.1± 0.1	-27.7± 0.2	-26.4± 0.2
B5	-14.8± 0.1	-17.4± 0.6	-16.5± 0.0	-13.8± 0.4	-8.1± 0.2	-16.1± 0.1	-17.7± 0.3	-14.8± 0.2	-20.0± 0.2	-18.3± 0.1	-8.3± 0.2	-18.0± 0.0	-17.8± 0.1
B6	-18.3± 0.1	-15.1± 0.1	-14.3± 0.1	-18.5± 0.1	-11.8± 0.2	-17.1± 0.5	-19.2± 0.1	-15.3± 0.1	-18.8± 0.2	-20.1± 0.1	-8.6± 1.3	-20.2± 0.1	-21.1± 0.0
B7	-15.3± 0.0	-15.3± 0.9	-13.9± 0.0	-14.3± 0.1	-9.8± 0.0	-15.6± 0.2	-17.4± 0.1	-12.9± 0.2	-18.5± 0.1	-18.5± 0.1	-7.4± 0.3	-18.3± 0.1	-19.0± 0.0
B8	-14.1± 0.1	-15.4± 0.7	-15.2± 0.0	-14.0± 0.3	-7.8± 0.3	-16.1± 0.2	-15.9± 0.0	-14.4± 0.1	-20.7± 0.3	-17.7± 0.0	-7.8± 0.5	-17.4± 0.2	-17.7± 0.0
B9	-18.1± 0.1	-24.2± 0.9	-21.2± 0.0	-21.7± 0.2	-16.8± 0.2	-21.2± 0.5	-24.1± 0.5	-21.5± 0.1	-27.4± 0.3	-27.0± 0.0	-16.8± 0.2	-25.2± 0.1	-25.1± 0.1
B10	-16.5± 0.0	-19.2± 0.0	-16.8± 0.1	-15.5± 0.3	-10.6± 0.4	-18.6± 0.1	-17.4± 0.1	-17.8± 0.2	NA	-20.9± 0.0	-12.2± 0.3	-19.7± 0.1	-21.4± 0.0
B11	-17.2± 0.1	-18.9± 0.1	-18.5± 0.0	-16.9± 0.6	-11.8± 0.5	-18.6± 0.1	-19.0± 0.1	-18.5± 0.1	NA	-21.9± 0.1	-11.6± 0.7	-20.9± 0.1	-22.1± 0.1
B12	-20.8± 0.1	-24.4± 0.0	-24.1± 0.0	-22.4± 0.4	NA± NA	-24.0± 0.1	-24.2± 0.1	-24.8± 0.2	-31.6± 0.3	-28.7± 0.1	-17.9± 1.6	-25.5± 0.0	-26.0± 0.1
Collembola													
cL1	-27.4± 0.1	-24.5± 1.0	-23.6± 0.1	-24.2± 0.0	-21.6± 0.2	-27.8± 0.1	-32.7± 0.2	-27.4± 0.1	-27.6± 0.1	-30.8± 0.1	-17.0± 0.0	-29.9± 0.1	-30.5± NA
cL2	-25.8± 0.1	-22.1± 1.0	-22.3± 0.1	-23.4± 0.1	-21.0± 0.4	-26.2± 0.1	-32.3± 0.2	-25.8± 0.2	-25.6± 0.2	-28.6± 0.2	-16.7± 0.2	-27.9± 0.1	-30.8± 0.2
cL3	-25.6± 0.1	-21.2± 0.1	-23.0± 0.0	-23.7± 0.1	-20.4± 0.3	-26.0± 0.2	-31.4± 0.3	-24.9± 0.1	-25.8± 0.1	-28.4± 0.0	-15.5± 0.2	-27.6± 0.1	-29.1± 0.3
cL4	-24.8± 0.1	-22.0± 0.2	-22.8± 0.1	-24.6± 0.1	-20.1± 0.2	-26.1± 0.1	-30.8± 0.1	-25.5± 0.0	-25.6± 0.1	-28.0± 0.0	-16.2± 0.2	-27.3± 0.1	-28.5± 0.0
cL5	-25.4± 0.1	-24.6± 1.0	-23.0± 0.0	-23.6± 0.1	-19.3± 0.4	-26.4± 0.0	-31.2± 0.1	-25.7± 0.0	-25.3± 0.1	-28.4± 0.1	-16.2± 0.2	-27.7± 0.0	-29.9± 0.1
Daphnia													
dL1	-29.8± 0.2	-28.2± 0.8	-27.2± 0.0	-22.7± 0.4	-22.5± 0.1	-30.9± 0.1	-35.6± 0.1	-26.5± 0.1	-28.6± 0.2	-36.0± 0.1	-15.8± 0.8	-33.4± 0.1	-33.3± 0.0
dL2	-32.4± 0.3	-30.6± 0.5	-30.5± 0.0	-24.6± 0.3	-25.9± 0.6	-33.8± 0.2	-38.3± 0.2	-29.2± 0.2	-31.5± 0.2	-38.8± 0.0	-20.8± 1.3	-35.9± 0.2	-37.4± 0.1
dL3	-26.6± 0.0	-27.0± 0.2	-25.7± 0.0	-21.5± 0.1	-21.7± 0.2	-29.6± 0.1	-34.0± 0.4	-25.3± 0.2	-27.8± 0.3	-34.2± 0.1	-16.8± 0.1	-31.6± 0.2	-32.5± 0.1
dL4	-36.1± 0.2	-32.1± 0.7	-31.6± 0.0	-31.6± 0.5	-29.0± 0.2	-34.6± 0.1	-39.7± 0.1	-31.0± 0.2	-33.1± 0.2	-40.2± 0.1	-21.8± 0.1	-37.0± 0.1	-38.8± 0.0
dL5	-32.6± 0.1	-28.8± 0.1	-29.0± 0.0	-23.6± 0.2	-22.6± 0.3	-32.2± 0.0	-37.1± 0.1	-28.6± 0.1	-30.8± 0.1	-37.4± 0.1	-20.6± 0.1	-34.8± 0.0	-35.8± 0.1
Fish													
ch	-14.3± 0.4	-17.8± 0.1	-14.6± 0.1	-7.0± 0.1	-9.6± 0.0	-18.4± 0.5	-25.4± 0.2	-16.9± 0.1	-25.4± 0.1	-26.7± 0.1	-10.1± 0.4	-25.8± 0.3	-22.1± 0.3
lg	-16.1± 0.1	-17.2± 1.4	-17.4± 0.3	-8.5± 0.1	-10.1± 0.1	-20.3± 0.0	-27.1± 0.1	-18.5± 0.1	-26.7± 0.1	-27.8± 0.1	-11.3± 0.6	-26.4± 0.1	-22.7± 0.3
xg	-16.5± 0.6	-16.0± 0.4	-18.2± 0.0	-5.9± 0.3	-7.4± 0.2	-20.1± 0.2	-27.7± 0.2	-18.8± 0.1	-25.6± 0.1	-27.1± 0.1	-8.7± 0.3	-26.0± 0.2	-23.2± 0.2
Fungi													
F1	-21.2± 0.1	-20.4± 0.1	-20.9± 0.0	-28.8± 0.3	-22.1± 0.3	-23.4± 0.1	-30.0± 0.2	-28.0± 0.4	-22.0± 0.2	-27.3± 0.0	-13.8± 0.5	-27.1± 0.1	-25.1± 0.1
F2	-16.0± 0.2	-19.7± 0.6	-20.7± 0.0	-22.8± 0.1	-20.6± 0.4	-22.3± 0.4	-27.2± 0.1	-26.5± 0.2	-24.5± 0.3	-26.5± 0.0	-11.9± 0.5	-26.6± 0.1	-21.7± 0.2
F3	-21.4± 0.1	-22.3± 0.1	-20.6± 0.1	-25.7± 0.1	NA± NA	-24.8± 0.1	-31.1± 0.5	-27.6± 0.7	-25.1± 0.4	-30.2± 0.4	-14.3± 0.1	-28.8± 0.1	-26.1± 0.0
F4	-18.3± 0.0	-21.9± 0.0	-21.3± 0.1	-23.2± 0.6	-22.9± 0.3	-22.7± 0.2	-29.5± 0.1	-28.5± 0.1	-25.2± 0.6	-27.9± 0.0	-12.2± 0.1	-27.5± 0.2	-26.8± 0.2

ID	Ala	Asx	Glx	Gly	His	Ile	Leu	Lys	Met	Phe	Thr	Tyr	Val
F8	-6.4± 0.1	-6.3± 0.1	-4.1± 0.0	-10.7± 0.1	-7.1± 0.4	-6.0± 0.0	-12.4± 0.1	-11.0± 0.2	-10.7± 0.3	-13.3± 0.0	-1.2± 0.1	-13.9± 0.1	-8.7± 0.1
F5	-5.5± 0.1	-6.6± 0.8	-4.9± 0.2	-8.7± 0.1	-8.3± 0.7	-8.5± 0.3	-14.7± 0.5	-12.7± 0.2	-10.3± 0.2	-14.5± 0.1	-1.9± 0.8	-14.3± 0.1	-10.3± 0.1
F6	-5.2± 0.0	-5.7± 0.5	-3.9± 0.0	-4.4± 0.2	-10.8± 0.3	-8.2± 0.4	-13.8± 0.4	-14.4± 0.6	-9.7± 0.1	-15.3± 0.1	-1.6± 0.5	-15.1± 0.1	-8.3± 0.3
F7	-4.7± 0.2	-4.5± 0.1	-4.2± 0.1	-8.1± 0.1	-9.2± 0.2	-9.5± 0.2	-17.3± 0.3	-14.2± 0.2	-12.0± 0.5	-15.5± 0.1	-3.3± 0.1	-14.6± 0.2	-10.0± 0.3
F9	-6.2± 0.1	-5.6± 0.2	-4.3± 0.0	-7.5± 0.1	-6.7± 0.3	-9.3± 0.1	-16.7± 0.1	-13.4± 0.4	-13.8± 0.5	-15.3± 0.1	-4.4± 0.0	-15.0± 0.1	-11.5± 0.0
Macroalgae													
R1	-9.0± 0.1	-10.9± 0.1	-14.3± 0.1	-4.2± 0.1	-12.5± 0.3	-17.3± 0.5	-20.2± 0.1	-10.9± 0.1	-15.3± 0.1	-19.2± 0.0	-6.4± 0.2	-18.9± 0.1	-19.2± 0.1
R2	-13.3± 0.2	-11.3± 0.6	-16.2± 0.0	-10.3± 0.3	NA	-15.4± 0.3	-19.5± 0.2	-13.0± 0.2	NA	-20.7± 0.1	-4.8± 0.6	-20.0± 0.1	-21.3± 0.3
R3	-17.3± 0.2	-16.4± 0.1	-18.6± 0.1	-14.7± 0.1	-19.4± 0.0	-21.7± 0.3	-25.8± 0.3	-16.4± 0.2	NA	-25.3± 0.1	-9.2± 0.2	-23.0± 0.1	-25.4± 0.2
R4	-13.4± 0.1	-11.1± 0.2	-13.8± 0.0	-16.4± 0.1	-12.9± 0.2	-17.0± 0.1	-21.9± 0.4	-14.9± 0.2	-21.6± 0.2	-23.7± 0.0	-11.0± 1.1	-21.9± 0.1	-19.8± 0.1
R5	-15.5± 0.1	-15.4± 0.0	-17.6± 0.6	-13.7± 0.0	NA	-19.5± 0.1	-22.5± 0.1	-16.4± 0.4	NA	-25.3± 0.1	-15.3± 0.1	-24.2± 0.0	-22.4± 0.0
R6	-11.4± 0.1	-11.4± 0.1	-14.8± 0.0	-9.8± 0.1	-14.2± NA	-16.5± 0.1	-21.0± 0.4	-13.1± 0.1	NA	-21.1± 0.1	-8.7± 0.1	-19.8± 0.1	-20.7± 0.2
R7	-12.1± 0.1	-12.4± 0.5	-15.4± 0.1	-9.9± 0.0	NA	-16.7± 0.2	-21.0± 0.3	-11.0± 0.1	-15.3± 0.1	-21.1± 0.3	-5.9± 0.3	-19.5± 0.4	-20.3± 0.0
R8	-14.7± 0.3	-14.5± 0.7	-16.1± 0.4	-14.5± 0.1	NA	-18.0± 0.1	-23.5± 0.0	-15.8± 0.4	-17.9± NA	-22.0± 0.0	-7.3± 1.1	-21.2± 0.3	-23.6± 0.0
R9	-11.9± 0.1	-13.2± 0.1	-15.6± 0.1	-10.7± 0.1	-16.4± 0.4	-20.4± 0.2	-23.4± 0.0	-13.3± 0.1	-17.3± 0.5	-22.5± 0.1	-6.8± 1.0	-20.1± 0.0	-20.9± 0.1
P1	-13.6± 0.2	-4.6± 0.3	-12.7± 0.1	-12.1± 0.1	-13.2± 0.3	-17.6± 0.2	-23.1± 0.2	-11.1± 0.1	-14.1± 0.2	-19.4± 0.1	-3.6± 0.2	-18.6± 0.1	-18.8± 0.1
P2	-10.3± 0.3	-1.9± 0.7	-11.4± 0.1	-9.6± 0.3	-12.6± 0.3	-15.2± 0.1	-21.5± 0.2	-9.1± 0.2	-13.0± 0.1	-19.1± 0.2	-1.2± 0.5	-18.2± 0.1	-17.0± 0.1
P3	-15.2± 0.3	-5.6± 0.7	-15.6± 0.1	-15.7± 0.3	NA	-19.6± 0.4	-24.8± 0.1	-12.7± 0.3	-16.0± 0.4	-22.2± 0.1	-5.5± 0.1	-21.1± 0.1	-20.7± 0.0
P4	-11.9± 0.3	-2.4± 0.2	-11.6± 0.1	-10.3± 0.0	-12.0± 0.9	-16.8± 1.4	-20.0± 0.1	-9.9± 0.1	-13.1± 0.3	-18.9± 0.1	-0.4± 0.3	-18.0± 0.2	-15.7± 0.1
P5	NA± 0.4	-9.2± 0.2	-18.0± 0.1	-17.2± 0.1	-16.7± 0.5	-21.4± 0.1	-26.1± 0.2	-14.1± 0.1	-17.5± 0.1	-23.9± 0.0	-6.4± 0.2	-22.2± 0.2	-22.5± 0.1
P6	-4.5± 0.0	-4.2± 1.0	-4.7± 0.0	-2.7± 0.2	NA	-6.3± 0.6	-13.3± 0.1	-1.7± 0.0	-7.2± 0.1	-12.8± 0.1	-2.6± 0.7	-12.2± 0.3	-10.4± 0.1
P7	-9.5± 0.2	-4.9± 0.1	-11.3± 0.0	-7.7± 0.2	-7.6± 0.1	-15.6± 0.1	-23.9± 0.4	-9.2± 0.4	-12.7± 0.5	-18.8± 0.1	-1.2± 0.5	-18.8± 0.1	-19.1± 0.1
P8	-10.4± 0.1	-9.0± 0.3	-12.0± 0.2	-6.9± 0.2	-14.9± 0.6	-19.5± 0.2	-25.5± 0.3	-10.7± 0.2	-17.8± 0.2	-20.4± 0.2	-0.5± 0.7	-21.9± 0.1	-19.6± 0.3
P9	-9.2± 0.2	-5.7± 1.1	-9.6± 0.0	-9.2± 0.3	NA	-10.9± 0.1	-18.3± 0.4	-8.2± 0.1	NA	-16.7± 0.1	0.9± 0.5	-17.0± 0.0	-14.5± 0.0
P10	-14.1± 0.1	-9.8± 0.1	-12.6± 0.1	-11.5± 0.1	NA	-16.9± 0.1	-23.6± 0.2	-10.8± 0.3	-14.3± 0.1	-23.6± 0.0	5.4± 4.6	-22.9± 0.1	-21.2± 0.2
P11	-5.2± 0.0	-5.5± 0.8	-5.3± 0.0	-1.9± 0.1	-5.3± 0.5	-7.3± 0.1	-14.7± 0.3	-4.5± 0.2	-7.8± NA	-12.7± NA	0.8± 0.3	-12.5± 0.0	-10.9± 0.2
P12	-14.4± 0.2	-8.7± 0.1	-17.1± 0.1	-16.2± 0.8	-15.1± 0.4	-17.6± 0.2	-25.2± 0.2	-9.1± 0.1	-14.7± 0.1	-21.2± 0.1	-8.6± 0.0	-20.4± 0.2	-22.4± 0.2
Microalgae													
C1	-13.6± 0.2	-12.8± 0.2	-23.7± 0.1	-15.3± 0.6	-23.5± 0.3	-27.7± 0.2	-27.8± 0.1	-18.0± 0.0	-23.3± 0.5	-24.9± 0.3	-10.4± 0.1	-23.7± 0.1	-24.0± 0.1
C2	-22.6± 0.1	-16.2± 0.1	-26.3± 0.0	-23.1± 0.2	NA	-28.9± 0.0	-36.0± 0.0	-23.8± 0.3	-23.9± 0.4	-34.6± 0.1	-19.5± 0.6	-35.0± 0.2	-32.8± 0.1
C3	-11.6± 0.1	-12.9± 1.0	-15.3± 0.0	-7.3± 0.2	NA	-17.3± 0.1	-23.3± 0.3	-13.4± 0.2	NA	-19.8± 0.1	-5.9± 1.0	-17.5± 0.2	-20.5± 0.2
C4	-13.1± 0.3	-12.2± 0.8	-18.9± 0.1	-11.3± 0.2	NA	-20.0± 0.1	-25.8± 0.0	-13.8± 0.3	NA	-24.0± 0.2	-10.1± 0.1	-22.1± 0.1	-24.2± 0.1
D1	-9.7± 0.1	-11.1± 0.4	-11.4± 0.1	-11.2± 0.2	NA	-10.2± 0.1	-17.3± 0.2	-8.8± 0.5	NA	-18.1± 0.3	-2.7± 0.8	-15.7± 0.1	-13.8± 0.1
D2	-7.4± 0.2	-9.1± 0.3	-7.5± 0.0	-12.0± 0.4	NA	-11.5± 0.1	-18.7± 0.0	-11.1± 0.2	NA	-19.6± 0.2	-2.0± 0.9	-16.0± 0.1	-16.8± 0.2
D3	-10.3± 0.1	-16.2± 1.0	-12.2± 0.0	-12.3± 0.1	NA	-16.5± 0.0	-22.1± 0.5	-15.8± 0.2	-15.0± 0.2	-20.6± 0.1	-7.0± 1.6	-19.4± 0.1	-19.7± 0.0
D4	-16.0± 0.1	-17.7± 0.1	-18.9± 0.0	-12.2± 0.1	NA	-16.1± 0.1	-25.5± 0.4	-15.2± 0.2	NA	-23.8± 0.1	-10.8± 0.3	-21.8± 0.1	-22.9± 0.1
D5	-1.3± 0.1	-6.6± 1.0	-7.4± 0.1	5.5± 0.2	NA	-10.4± 0.1	-19.3± 0.1	-8.5± 0.2	-8.2± 0.4	-16.0± 0.4	-4.6± 0.5	-14.4± 0.2	-15.5± 0.1

ID	Ala	Asx	Glx	Gly	His	Ile	Leu	Lys	Met	Phe	Thr	Tyr	Val
H1	-16.3± 0.0	-15.6± 0.1	-15.9± 0.1	-13.5± 0.0	-17.8± 0.3	-18.3± 0.6	-27.2± 0.0	-15.7± 0.1	-17.9± 0.1	-23.2± 0.0	-9.1± 0.1	-22.3± 0.0	-24.9± 0.0
H2	-13.1± 0.1	-13.1± 1.0	-19.7± 0.1	-19.0± 0.1	NA	-19.2± 0.2	-23.8± 0.1	-14.5± 0.1	NA	-24.6± 0.0	-8.6± 0.6	-22.6± 0.1	-21.1± 0.3
H3	-16.4± 0.1	-13.3± 0.2	-16.7± 0.1	-19.6± 0.1	NA	-18.3± 0.1	-31.4± 0.1	-15.4± 0.1	-19.9± 0.2	-25.0± 0.0	-8.9± 0.2	-22.7± 0.1	-26.8± 0.1
H4	-11.2± 0.0	-13.7± 0.9	-15.9± 0.0	-11.8± 0.1	NA	-13.8± 0.1	-20.5± 0.1	-12.0± 0.4	NA	-21.6± 0.1	-6.8± 0.3	-19.6± 0.1	-18.8± 0.0
K1	-12.1± 0.5	-13.0± 0.7	-15.6± 0.1	-7.1± 0.6	NA	-15.8± 1.3	-23.7± 0.3	-13.0± 0.3	-16.9± 0.3	-19.7± 0.1	-6.7± 0.5	-16.8± 0.2	-19.2± 0.5
K2	-12.7± 0.1	-12.3± 0.9	-14.3± 0.1	-7.9± 0.1	NA	-15.3± 0.5	-23.3± 0.1	-12.2± 0.0	-15.5± 0.4	-21.4± 0.0	-2.2± 0.8	-19.4± 0.1	-20.6± 0.1
K3	-4.1± 0.0	-5.7± 0.0	-10.0± 0.1	0.2± 0.1	NA	-10.4± 0.1	-15.7± 0.1	-5.2± 0.1	NA	-15.9± 0.1	-8.1± 0.9	-14.2± 0.0	-13.0± 0.1
K4	-5.5± 0.1	-7.5± 0.1	-10.8± 0.1	-6.6± 0.1	NA	-11.4± 0.0	-16.7± 0.1	-7.7± 0.3	NA	-15.7± 0.1	-4.2± 0.3	-12.5± 0.1	-15.3± 0.0
K5	-13.8± 0.1	-14.0± 0.4	-17.7± 0.1	-13.8± 0.1	NA	-16.3± 0.2	-23.2± 0.3	-13.9± 0.4	NA	-22.2± 0.0	-6.4± 0.3	-20.3± 0.2	-20.4± 0.1
K6	-9.6± 0.2	-13.4± 0.1	-12.1± 0.0	-10.7± 0.4	NA	-11.4± 0.5	-18.4± 0.1	-10.5± 0.3	NA	-18.4± 0.1	-7.1± 0.3	-16.8± 0.1	-15.9± 0.1
N1	-17.4± 0.1	-18.9± 0.0	-20.3± 0.1	-21.7± 0.4	-19.2± 0.6	-21.0± 0.1	-29.7± 0.3	-18.1± 0.0	-21.7± 0.6	-27.0± 0.1	-11.0± 0.1	-24.8± 0.2	-26.4± 0.5
N2	-17.4± 0.2	-18.2± 0.1	-20.3± 0.1	-21.9± 0.3	-20.8± 0.2	-20.6± 0.1	-29.3± 0.3	-18.0± 0.2	-19.2± 0.6	-26.8± 0.1	-11.2± 0.1	-24.5± 0.2	-27.2± 0.2
N3	-17.4± 0.2	-17.8± 0.2	-20.4± 0.0	-21.8± 0.6	-21.5± 0.2	-19.8± 0.1	-29.4± 0.2	-17.9± 0.2	-21.2± 0.4	-26.9± 0.1	-10.7± 0.1	-24.7± 0.2	-27.1± 0.1
X1	-17.2± 0.1	-16.5± 0.1	-24.5± 0.3	-14.7± 0.1	-25.4± 0.7	-26.7± 0.0	-35.1± 0.0	-20.7± 0.1	-27.3± 0.2	-31.9± 0.1	-13.6± 0.1	-29.7± 0.1	-30.2± 0.1
X2	-16.0± 0.0	-17.4± 0.9	-21.4± 0.0	-14.2± 0.2	NA	-22.8± 0.2	-32.7± 0.0	-19.2± 0.1	NA	-30.0± 0.1	-10.7± 0.8	-27.8± 0.1	-28.0± 0.1
X3	-16.3± 0.0	-18.3± 1.1	-18.9± 0.0	-14.5± 0.4	NA	-19.3± 0.1	-25.2± 0.1	-16.4± 0.3	NA	-25.5± 0.1	-9.6± 0.5	-23.3± 0.1	-23.9± 0.2
X4	-3.2± 0.2	-6.1± 0.1	-9.9± 0.4	-8.2± 0.0	NA	-11.1± 0.1	-18.2± 0.0	-6.2± 0.2	NA	-16.1± 0.0	-1.3± 0.2	-13.0± 0.1	-14.4± 0.1
Y1	-11.1± 0.1	-12.2± 0.1	-15.9± 0.1	-11.8± 0.1	NA	-11.8± 0.5	-19.3± 0.3	-11.4± 0.2	NA	-19.5± 0.1	-4.2± 0.3	-17.0± 0.1	-17.1± 0.1
Mussels													
gav	-13.1± 0.1	-15.9± 2.1	-13.2± 0.0	-11.9± 0.1	-16.9± 0.8	-18.6± 0.1	-25.1± 0.1	-14.3± 0.1	-17.3± 0.3	-24.2± 0.1	-12.9± 0.2	-22.7± 0.1	-23.2± 0.1
sc	-12.3± 0.0	-14.2± 0.1	-12.0± 0.1	-6.8± 0.0	-13.1± 0.2	-16.8± 0.0	-23.4± 0.0	-13.1± 0.0	-15.8± 0.1	-22.5± 0.1	-11.0± 0.3	-21.3± 0.1	-21.5± 0.1
Plants													
T1	-27.2± 0.2	-25.7± 1.3	-29.4± 0.1	-24.2± 0.1	-24.9± 0.2	-29.2± 0.1	-38.4± 0.2	-25.6± 0.2	NA	-32.5± 0.4	-15.7± 0.3	-30.3± 0.1	-37.1± 0.5
T2	-24.6± 0.0	-25.5± 0.0	-29.1± 0.0	-24.5± 0.0	-32.4± 0.8	-30.7± 0.1	-39.7± 0.4	-27.9± 0.1	NA	-33.9± 0.1	-16.1± 2.3	-31.5± 0.0	-36.1± 0.2
T3	-25.1± 0.1	-19.9± 0.5	-23.7± 0.1	-20.1± 0.1	-21.5± 0.6	-24.2± 0.3	-34.4± 0.2	-22.5± 0.1	NA	-27.0± 0.1	-12.6± 0.3	-26.0± 0.3	-31.4± 0.3
T4	-27.0± 0.0	-23.0± 0.6	-26.5± 0.1	-23.3± 0.1	-26.0± 0.3	-27.4± 0.5	-35.3± 0.1	-23.9± 0.4	-28.9± 0.3	-28.8± 0.0	-15.8± 0.1	-28.6± 0.1	-33.3± 0.1
T5	-25.6± 0.0	-22.7± 0.1	-26.0± 0.1	-18.7± 0.1	-22.9± 0.2	-26.8± 0.4	-33.5± 0.2	-22.5± 0.1	NA	-27.7± 0.0	-15.3± 0.1	-26.0± 0.0	-31.6± 0.1
T6	-26.9± 0.0	-22.0± 0.1	-26.4± 0.1	-19.3± 0.2	-27.3± 0.1	-27.1± 0.1	-34.4± 0.1	-24.9± 0.1	-25.6± 0.4	-28.9± 0.2	-9.9± 0.7	-29.3± 0.0	-32.0± 0.1
T7	-27.0± 0.1	-22.0± 0.6	-25.7± 0.1	-21.9± 0.2	-27.0± 0.3	-27.5± 0.2	-33.6± 0.1	-23.5± 0.4	-32.2± 0.3	-28.7± 0.1	-12.6± 1.0	-28.3± 0.1	-32.2± 0.1
T8	-26.2± 0.2	-19.1± 0.1	-25.9± 0.0	-20.2± 0.1	-30.1± 0.2	-25.2± 0.1	-35.2± 0.1	-23.9± 0.1	-33.0± 0.3	-27.8± 0.1	-8.6± 0.5	-28.1± 0.1	-30.6± 0.1
T9	-26.7± 0.0	-22.9± 0.1	-26.8± 0.0	-21.7± 0.1	-25.1± 0.7	-27.8± 0.1	-35.4± 0.4	-24.3± 0.3	-26.8± 0.7	-28.9± 0.1	-15.4± 0.0	-29.0± 0.1	-32.6± 0.1
T10	-24.7± 0.1	-19.4± 0.7	-23.4± 0.0	-19.4± 0.4	-23.4± 0.9	-25.1± 0.3	-34.5± 0.4	-23.2± 0.4	-28.6± 0.2	-26.4± 0.1	-11.5± 0.1	-27.0± 0.2	-30.5± 0.5
T11	-25.2± 0.4	-18.8± 0.1	-23.6± 0.0	-18.6± 0.2	-25.4± NA	-23.2± 0.1	-33.0± 0.1	-21.8± 0.1	-24.5± NA	-27.0± 0.1	-4.4± 0.5	-27.3± 0.1	-30.0± 0.2
T12	-27.3± 0.1	-24.1± 0.0	-26.1± 0.1	-21.1± 0.2	NA	-27.6± 0.1	-37.2± 0.1	-25.6± 0.2	NA	-29.9± 0.1	-18.1± 0.4	-28.7± 0.0	-34.1± 0.2
Seagrasses													
S1	-11.4± 0.1	-10.1± 0.2	-12.7± 0.3	-6.5± 0.1	NA	-13.9± 0.0	-19.5± 0.2	-9.5± 0.2	NA	-14.1± 0.1	-5.6± 0.2	-17.0± 0.1	-17.3± 0.0
S2	-12.7± 0.1	-12.2± 0.0	-15.5± 0.0	NA	NA	-14.5± 0.1	-20.3± 0.1	-10.3± 1.2	NA	-15.1± 0.0	-5.7± 0.1	-18.2± 0.4	-18.3± 0.1
S3	-13.9± 0.2	-12.7± 0.2	-15.9± 0.1	-10.3± 0.4	NA	-14.5± 0.1	-21.7± 0.0	-11.2± 0.6	NA	-15.6± 0.3	-7.5± 0.2	-18.6± 0.1	-19.4± 0.0

ID	Ala	Asx	Glx	Gly	His	Ile	Leu	Lys	Met	Phe	Thr	Tyr	Val
S4	-14.4± 0.2	-13.6± 0.4	-16.4± 0.1	-9.2± 0.3	NA	-14.9± 0.1	-22.4± 0.5	-11.5± 0.2	NA	-17.6± 0.1	-6.6± 0.3	-18.8± 0.0	-20.1± 0.1
S5	-13.8± 0.1	-14.0± 0.0	-16.9± 0.1	-9.1± 0.3	NA	-15.1± 0.1	-22.5± 0.1	-11.5± 0.3	NA	-16.5± 0.3	-5.7± 0.6	-18.8± 0.0	-19.5± 0.0
S6	-12.0± 0.2	-15.2± 0.8	-15.2± 0.0	-6.7± 0.3	NA	-17.5± 0.1	-21.4± 0.1	-12.7± 0.1	NA	-17.0± 0.1	-12.2± 0.2	-17.1± 0.2	-20.1± 0.0
S7	-16.7± 0.1	-17.7± 0.9	-17.7± 0.1	-9.3± 0.0	NA	-19.8± 0.5	-23.1± 0.2	-13.0± 0.2	NA	-17.6± 0.1	NA	-19.3± 0.1	-21.0± 0.1
Seston													
pL1	-27.2± 0.0	-24.6± 0.2	-28.6± 0.0	-24.6± 0.0	NA	-33.5± 0.2	-37.5± 0.0	-26.6± 0.2	-28.9± 0.6	-36.1± 0.1	NA	-33.1± 0.0	-35.7± 0.3
pL2	-31.6± 0.5	-28.8± 0.1	-34.1± 0.1	-30.7± 1.1	NA	-39.0± 0.3	-42.6± 0.3	-29.6± 0.3	-31.1± 0.4	-41.3± 0.1	-23.5± 4.5	-36.3± 0.1	-41.7± 0.2
Soil													
sL1	-25.2± 0.2	-20.5± 0.2	-25.1± 0.1	-19.7± 0.1	NA	-28.2± 0.1	-33.6± 0.1	-25.3± 0.6	-28.0± 0.6	-29.5± 0.1	-16.6± 0.2	-27.9± 0.1	-32.6± 0.3
sL2	-23.3± 0.1	-16.3± 0.6	-22.8± 0.0	-16.5± 0.1	NA	-26.7± 0.1	-31.9± 0.1	-23.2± 0.2	-25.5± 0.2	-28.4± 0.1	-17.8± 0.5	-27.4± 0.1	-30.9± 0.0
sL4	-22.7± 0.1	-21.0± 0.1	-22.4± 0.1	-16.3± 0.1	NA	-24.6± 0.1	-30.9± 0.1	-23.0± 0.3	NA	-27.2± 0.1	-16.2± 0.1	-26.4± 0.3	-30.0± 0.1
sL5	-21.0± 0.1	-19.7± 0.6	-21.8± 0.2	-13.7± 0.2	NA	-24.2± 0.1	-29.0± 0.6	-21.2± 0.2	-25.5± 0.4	-26.4± 0.1	-15.9± 0.5	-25.0± 0.2	-28.1± 0.5
