# Supplementary material for: Tracing Carbon Sources through Aquatic and Terrestrial Food Webs Using Amino Acid Stable Isotope Fingerprinting
Source: PLoS One. 2013 Sep 17;8(9):e73441. doi: 10.1371/journal.pone.0073441 (PMC3775739; doi:10.1371/journal.pone.0073441)
Supplement: Table S4 — Principal component analysis output for algal, bacterial, fungal and plant samples ( Figure 1 ). (PDF) [file pone.0073441.s005.pdf]

Supporting Table S4

Principal component analysis output for Fig. 1.

Importance of components

	PC1	PC2	PC3	PC4	PC5	PC6	PC7	PC8	PC9	PC10
Eigenvalue	3.5134	2.3968	1.506	0.87973	0.79135	0.59113	0.55969	0.33367	0.18363	0.11818
Cumulative proportion	0.3231	0.5435	0.682	0.76294	0.83572	0.89009	0.94156	0.97224	0.98913	1

Vector scores

	PC1	PC2	PC3	PC4	PC5	PC6
Ala	0.08	1.09	-0.93	0.23	-0.21	0.54
Asx	-1.20	0.62	0.50	-0.15	0.31	0.47
Glx	0.52	1.03	0.72	0.38	-0.49	0.05
Gly	-0.96	-0.22	-0.89	0.76	-0.18	-0.61
Ile	1.12	-0.10	0.29	-0.40	-1.00	-0.27
Leu	1.39	-0.13	-0.35	-0.44	0.52	-0.19
Lys	-1.02	-0.48	-0.51	-1.01	-0.18	0.02
Phe	0.49	-1.34	0.25	0.52	0.25	0.08
Thr	-0.86	-0.23	1.14	0.02	0.12	-0.21
Tyr	0.54	-1.26	-0.09	0.22	-0.28	0.78
Val	1.34	0.63	-0.01	-0.01	0.60	-0.11

Sample scores

ID	PC1	PC2
B1	0.86	0.14
B10	0.90	-0.62
B11	0.80	-0.57
B12	0.96	-0.28
B2	1.34	-0.63
B3	1.31	0.11
B4	0.62	-0.07
B5	0.81	-0.77
B6	0.62	-0.46
B7	0.58	-0.56
B8	0.78	-0.66
B9	0.96	-0.06
C1	-0.82	0.06
C2	-0.84	1.13
C3	-0.31	-0.33
C4	-0.65	0.14
D1	0.46	0.06
D2	0.21	0.79
D3	0.37	0.36
D4	0.12	-0.29
D5	-0.55	0.78
F1	0.82	1.34
F2	0.83	0.73
F3	0.68	0.68
F4	0.68	0.45
F5	0.82	0.89
F6	0.96	0.28
F7	0.68	1.29
F8	1.12	0.76

ID	PC1	PC2
F9	0.67	1.03
H1	-0.21	-0.06
H2	-0.05	0.33
H3	-0.40	0.10
H4	0.17	0.13
K1	-0.15	-0.44
K2	-0.48	0.05
K3	-0.18	0.40
K4	0.02	-0.14
K5	0.01	-0.13
K6	0.56	0.05
N1	0.03	0.05
N2	0.00	-0.01
N3	-0.02	0.02
P1	-0.65	0.20
P10	-1.07	0.78
P11	0.01	0.23
P12	-0.58	-0.20
P2	-0.83	0.64
P3	-0.70	0.34
P4	-0.71	0.52
P6	0.10	0.34
P7	-0.97	0.52
P8	-1.12	0.80
P9	-0.20	0.42
R1	-0.52	0.16
R2	-0.23	-0.13
R3	-0.33	-0.09
R4	0.24	0.80

ID	PC1	PC2
R5	0.19	0.50
R6	-0.15	0.22
R7	-0.31	-0.01
R8	-0.05	-0.10
R9	-0.43	0.22
S1	-0.09	-0.58
S3	0.07	-0.87
S4	-0.08	-0.78
S5	-0.07	-0.90
S6	0.14	-0.77
T1	-0.52	-0.90
T10	-0.40	-0.73
T11	-0.66	-0.70
T12	-0.22	-0.88
T2	-0.52	-0.25
T3	-0.32	-0.93
T4	-0.23	-0.98
T5	-0.28	-1.21
T6	-0.55	-0.77
T7	-0.36	-0.89
T8	-0.57	-0.78
T9	-0.31	-0.89
X1	-1.14	0.79
X2	-0.77	0.76
X3	-0.01	0.01
X4	-0.19	0.28
Y1	0.29	-0.29
