# Supplementary material for: Tracing Carbon Sources through Aquatic and Terrestrial Food Webs Using Amino Acid Stable Isotope Fingerprinting
Source: PLoS One. 2013 Sep 17;8(9):e73441. doi: 10.1371/journal.pone.0073441 (PMC3775739; doi:10.1371/journal.pone.0073441)
Supplement: Table S5 — ANOVA comparison of δ13CAAn values. (PDF) [file pone.0073441.s006.pdf]

Supporting Table S5

Comparison of $\delta^{13}C_{AAn}$ values (mean ± standard deviation) between groups with the number of replicates (n) in the rightmost column. The subscript letters denote significant differences between groups at 95% confidence levels with analysis of variance tests.

	Ala	Asx	Glx	Gly	Ile	Leu	Lys	Phe	Thr	Tyr	Val	n
Overall comparison												
Algae	3.4±1.7[a]	4.1±2.9[a]	0.6±1.5[a]	4.0±3.2[a]	-1.1±2.0[a]	-7.6±1.9[a]	2.9±1.2[a]	-6.1±0.9[a]	9.1±2.9[a]	-4.5±1.4[a]	-4.9±1.2[a]	47
Bacteria	1.6±1.6[b]	-0.7±1.4[b]	0.5±1.5[a]	0.5±1.4[b]	0.2±0.8[ab]	-0.8±1.1[b]	0.8±1.2[b]	-3.8±1.2[b]	6.9±1.2[b]	-2.8±0.8[b]	-2.4±1.4[b]	12
Fungi	4.0±1.2[a]	3.1±1.1[a]	4.0±1.4[b]	0.1±2.6[b]	0.7±0.8[b]	-5.8±1.1[c]	-4.0±1.1[c]	-5.0±1.0[a]	8.5±2.1[ab]	-4.7±1.0[a]	-0.9±1.1[c]	9
Plants	-0.2±1.7[b]	3.8±0.8[a]	-0.2±0.7[a]	4.8±1.0[a]	-0.9±0.6[ab]	-9.5±0.9[d]	1.8±0.8[d]	-3.1±0.8[c]	12.9±2.7[c]	-2.5±1.0[b]	-6.8±0.7[d]	12
Algal comparison												
Microalgae	4.2±1.6[a]	3.2±2.6[a]	0.3±1.7[a]	4.1±3.8[a]	-0.5±2.0[a]	-7.8±1.9[a]	2.7±1.0[a]	-6.1±1.0[a]	8.7±2.3[a]	-4.0±1.2[a]	-4.9±1.2[a]	27
Phaeophyceae	1.8±1.1[b]	6.9±3.2[b]	1.3±0.9[a]	3.1±2.2[a]	-2.3±1.9[b]	-8.7±1.5[a]	3.7±1.4[b]	-6.2±1.1[a]	11.0±4.0[a]	-5.8±1.3[b]	-4.8±1.3[a]	11
Rhodophyta	3.2±1.0[ab]	3.5±0.9[a]	0.6±1.2[a]	4.8±2.4[a]	-1.6±1.4[ab]	-5.7±1.1[b]	2.6±0.7[ab]	-5.9±0.6[a]	8.0±2.4[a]	-4.5±0.6[ab]	-5.1±0.6[a]	9
Microalgae												
Chlorophytes	3.5±1.3[a]	2.2±1.3[a]	-0.3±0.8[a]	5.5±2.9[a]	-0.3±1.1[a]	-7.0±1.4[a]	2.7±0.7[a]	-5.8±0.7[a]	7.4±3.9[a]	-3.6±1.2[a]	-4.3±1.1[a]	6
Chrysophytes	5.5±1.7[a]	4.1±2.6[ab]	0.0±0.5[a]	5.7±3.3[a]	-1.4±1.2[ab]	-9.2±2.5[a]	3.0±0.4[a]	-7.2±1.1[a]	9.8±1.1[a]	-4.8±1.4[a]	-5.5±1.0[a]	4
Cyanobacteria	4.8±1.3[a]	6.5±3.7[b]	-1.0±1.9[a]	5.7±1.6[a]	-3.5±2.8[b]	-8.2±0.5[a]	2.8±1.1[a]	-5.8±1.3[a]	8.6±1.1[a]	-4.6±2.3[a]	-5.3±1.1[a]	4
Diatoms	4.2±1.8[a]	1.9±1.5[a]	1.6±1.7[a]	2.9±5.2[a]	0.6±1.2[a]	-7.6±1.5[a]	2.2±1.5[a]	-6.0±0.9[a]	8.9±2.1[a]	-3.8±0.7[a]	-4.8±1.4[a]	8
Haptophytes	3.5±1.3[a]	3.9±2.2[ab]	0.7±2.3[a]	1.8±2.7[a]	-0.4±1.1[a]	-7.9±3.1[a]	3.3±0.6[a]	-5.8±0.8[a]	9.4±1.0[a]	-4.0±0.6[a]	-5.1±2.1[a]	4
