# Supplementary material for: Tracing Carbon Sources through Aquatic and Terrestrial Food Webs Using Amino Acid Stable Isotope Fingerprinting
Source: PLoS One. 2013 Sep 17;8(9):e73441. doi: 10.1371/journal.pone.0073441 (PMC3775739; doi:10.1371/journal.pone.0073441)
Supplement: Table S6 — Linear discriminant analysis output for algal, bacterial, fungal and terrestrial plant samples ( Figure 2 ). (PDF) [file pone.0073441.s007.pdf]

Supporting Table S6

Linear discriminant function analysis output for Fig. 2. According to the MANOVA test, algae, bacteria, fungi and plants are significantly different: Pillai Trace = 3.16, $F_{50,345}$ = 11.8, P<0.0001.

Coefficients of linear discriminants

	LD1	LD2	LD3
Ile	-0.09	0.16	0.14
Leu	0.11	-0.59	-0.46
Lys	0.74	-0.13	0.13
Phe	-0.25	0.45	-0.44
Thr	-0.02	0.05	0.00
Val	-0.44	0.02	0.68

Proportion of trace

	LD1	LD2	LD3
	0.55	0.28	0.17

Posterior probabilities with cross validation of the classifier samples.

ID	Actual	Predicted (% probability)			
		Algae	Bacteria	Fungi	Plants
C1	Algae	**94.7**	4.2	0	1.2
C2	Algae	**100**	0	0	0
C3	Algae	**98.1**	0	0	1.9
C4	Algae	**100**	0	0	0
D1	Algae	**100**	0	0	0
D2	Algae	**99.9**	0.1	0	0
D3	Algae	**84.0**	12	0	3.9
D4	Algae	**100**	0	0	0
D5	Algae	**100**	0	0	0
H1	Algae	**91.7**	0	0	8.3
H2	Algae	**100**	0	0	0
H3	Algae	**60.9**	0	0	39.1
H4	Algae	**100**	0	0	0
K1	Algae	**99.0**	0	0	1
K2	Algae	**100**	0	0	0
K3	Algae	**100**	0	0	0
K4	Algae	**100**	0	0	0
K5	Algae	**100**	0	0	0
K6	Algae	**100**	0	0	0
N1	Algae	**99.8**	0	0	0.2
N2	Algae	**99.6**	0	0	0.4
N3	Algae	**99.6**	0	0	0.4
P1	Algae	**100**	0	0	0
P10	Algae	**100**	0	0	0
P11	Algae	**100**	0	0	0
P12	Algae	**100**	0	0	0
P2	Algae	**100**	0	0	0
P3	Algae	**100**	0	0	0
P4	Algae	**100**	0	0	0
P6	Algae	**100**	0	0	0
P7	Algae	**100**	0	0	0
P8	Algae	**100**	0	0	0
P9	Algae	**100**	0	0	0
R1	Algae	**99.8**	0.2	0	0
R2	Algae	45.9	**54.1**	0	0
R3	Algae	**99.8**	0.2	0	0
R4	Algae	**99.4**	0.6	0	0
R5	Algae	**61.5**	38.5	0	0

(Table S5 continued)		Predicted (% probability)			
ID	Actual	Algae	Bacteria	Fungi	Plants
R6	Algae	**99.2**	0.8	0	0
R7	Algae	**100**	0	0	0
R8	Algae	**88.2**	5.5	0	6.3
R9	Algae	**100**	0	0	0
X1	Algae	**100**	0	0	0
X2	Algae	**100**	0	0	0
X3	Algae	**99.9**	0.1	0	0
X4	Algae	**100**	0	0	0
Y1	Algae	**100**	0	0	0
B1	Bacteria	13.3	**86.7**	0	0
B10	Bacteria	0	**100**	0	0
B11	Bacteria	0	**100**	0	0
B12	Bacteria	0	**100**	0	0
B2	Bacteria	0	**100**	0	0
B3	Bacteria	0	**100**	0	0
B4	Bacteria	6.1	**93.9**	0	0
B5	Bacteria	0.5	**99.5**	0	0
B6	Bacteria	1.2	**98.8**	0	0
B7	Bacteria	5.1	**94.9**	0	0
B8	Bacteria	0	**100**	0	0
B9	Bacteria	0.4	**99.6**	0	0
F1	Fungi	0	0	**100**	0
F2	Fungi	0	0	**100**	0
F3	Fungi	0	0	**100**	0
F4	Fungi	0	0	**100**	0
F5	Fungi	0	0	**100**	0
F6	Fungi	0	0	**100**	0
F7	Fungi	0	0	**100**	0
F8	Fungi	0	0	**100**	0
F9	Fungi	0	0	**100**	0
T1	Plants	0.4	0	0	**99.6**
T10	Plants	0	0	0	**100**
T11	Plants	0.1	0	0	**99.9**
T12	Plants	0	0	0	**100**
T2	Plants	0.3	0	0	**99.7**
T3	Plants	0	0	0	**100**
T4	Plants	0.1	0	0	**99.9**
T5	Plants	0.7	0	0	**99.3**
T6	Plants	0	0	0	**100**
T7	Plants	0.6	0	0	**99.4**
T8	Plants	0	0	0	**100**
T9	Plants	0.1	0	0	**99.9**
