# Supplementary material for: Tracing Carbon Sources through Aquatic and Terrestrial Food Webs Using Amino Acid Stable Isotope Fingerprinting
Source: PLoS One. 2013 Sep 17;8(9):e73441. doi: 10.1371/journal.pone.0073441 (PMC3775739; doi:10.1371/journal.pone.0073441)
Supplement: Table S7 — Linear discriminant function analysis output of aquatic primary producers ( Figure 3 ). (PDF) [file pone.0073441.s008.pdf]

Supporting Table S7

Linear discriminant analysis output for Fig. 3. According to the MANOVA test, Microalgae, Phaeophyceae, Rhodophyta and Seagrass are significantly different: Pillai Trace=1.44, $F_{12,147}$=11.24 P<0.001.

Coefficients of linear discriminants

	LD1	LD2	LD3
Ile	0.12	-0.08	0.46
Leu	-0.49	-0.75	-0.56
Lys	0.39	0.20	-0.64
Phe	-0.78	0.43	0.21
Val	0.77	0.38	0.40

Proportion of trace

	LD1	LD2	LD3
	0.51	0.35	0.14

Posterior probabilities of the classifier samples.

ID	Actual	Predicted (% probability)			
		Microalgae	Phaeophyceae	Rhodophyta	Seagrass
C1	Microalgae	1.3	9.6	**55.8**	33.4
C2	Microalgae	**97.3**	0.1	2.7	0.0
C3	Microalgae	**73.9**	2.0	3.9	20.3
C4	Microalgae	34.4	6.5	**59.0**	0.0
D1	Microalgae	**85.2**	14.8	0.0	0.0
D2	Microalgae	**96.6**	0.3	3.1	0.0
D3	Microalgae	**84.4**	0.0	9.3	6.3
D4	Microalgae	**99.6**	0.1	0.3	0.0
D5	Microalgae	**71.3**	28.5	0.0	0.2
H1	Microalgae	**91.6**	0.2	2.9	5.2
H2	Microalgae	**87.3**	4.0	8.6	0.0
H3	Microalgae	**78.8**	21.1	0.0	0.1
H4	Microalgae	**89.5**	0.6	9.9	0.0
K1	Microalgae	**92.4**	7.0	0.0	0.6
K2	Microalgae	**94.5**	4.8	0.7	0.0
K3	Microalgae	2.0	**97.9**	0.1	0.0
K4	Microalgae	40.5	11.2	**46.0**	2.3
K5	Microalgae	**98.3**	0.7	1.0	0.0
K6	Microalgae	**97.9**	1.1	1.0	0.0
N1	Microalgae	**99.5**	0.2	0.2	0.0
N2	Microalgae	**95.8**	0.1	4.2	0.0
N3	Microalgae	**98.9**	0.1	1.0	0.0
X1	Microalgae	**90.5**	9.5	0.0	0.0
X2	Microalgae	**98.0**	2.0	0.0	0.0
X3	Microalgae	**51.1**	0.0	48.9	0.0
X4	Microalgae	2.3	**97.7**	0.0	0.0
Y1	Microalgae	**98.2**	0.3	1.5	0.0
P1	Phaeophyceae	31.9	**67.8**	0.1	0.2
P10	Phaeophyceae	**59.2**	35.4	5.4	0.0
P11	Phaeophyceae	29.2	**70.8**	0.0	0.0
P12	Phaeophyceae	3.8	**95.8**	0.4	0.0
P2	Phaeophyceae	6.9	**93.0**	0.0	0.0
P3	Phaeophyceae	39.2	**60.6**	0.2	0.0
P4	Phaeophyceae	22.3	**77.5**	0.2	0.0
P6	Phaeophyceae	1.7	**98.3**	0.0	0.0
P7	Phaeophyceae	4.7	**95.3**	0.0	0.0

(Table S7 continued)		Predicted (% probability)			
ID	Actual	Microalgae	Phaeophyceae	Rhodophyta	Seagrass
P8	Phaeophyceae	0.8	**99.2**	0.0	0.0
P9	Phaeophyceae	**75.5**	24.5	0.0	0.0
R1	Rhodophyta	6.6	1.1	**91.5**	0.8
R2	Rhodophyta	0.0	0.0	**99.9**	0.0
R3	Rhodophyta	3.5	0.0	**96.4**	0.0
R4	Rhodophyta	**98.9**	0.2	1.0	0.0
R5	Rhodophyta	4.3	0.0	**95.7**	0.0
R6	Rhodophyta	7.4	0.0	**92.5**	0.0
R7	Rhodophyta	12.3	1.6	**86.1**	0.0
R8	Rhodophyta	7.6	0.0	**79.7**	12.7
R9	Rhodophyta	**65.2**	20.1	14.6	0.0
S1	Seagrass	0.0	0.0	0.0	**100.0**
S2	Seagrass	0.1	0.0	0.0	**99.9**
S3	Seagrass	0.0	0.0	0.0	**100.0**
S4	Seagrass	8.0	0.2	0.5	**91.3**
S5	Seagrass	0.6	0.0	0.0	**99.4**
S6	Seagrass	0.0	0.0	0.2	**99.8**
S7	Seagrass	0.0	0.0	0.0	**99.9**
