# Supplementary material for: Tracing Carbon Sources through Aquatic and Terrestrial Food Webs Using Amino Acid Stable Isotope Fingerprinting
Source: PLoS One. 2013 Sep 17;8(9):e73441. doi: 10.1371/journal.pone.0073441 (PMC3775739; doi:10.1371/journal.pone.0073441)
Supplement: Table S8 — Linear discriminant function analysis output for amino acid origins of consumers ( Figure 5 ). (PDF) [file pone.0073441.s009.pdf]

Supporting Table S8 (for Figure 5a)

Linear discriminant analysis output for Figure 5a. According to the MANOVA test, Bacteria, Microalgae, and Plants are significantly different: Pillai Trace=1.57, $F_{10,90}$=33.2, P<0.001.

Coefficients of linear discriminants.

	LD1	LD2
Ile	0.07	0.00
Leu	-0.83	0.04
Lys	0.28	-0.50
Phe	0.08	0.62
Thr	0.06	0.08
Val	0.39	-0.37

Proportion of trace.

LD1	LD2
0.63	0.37

Posterior probabilities of the classifier samples.

ID	Actual	Predicted (% probability)		
		Bacteria	Microalgae	Plants
B1	Bacteria	**99.8**	0.2	0.0
B10	Bacteria	**100.0**	0.0	0.0
B11	Bacteria	**100.0**	0.0	0.0
B12	Bacteria	**100.0**	0.0	0.0
B2	Bacteria	**100.0**	0.0	0.0
B3	Bacteria	**100.0**	0.0	0.0
B4	Bacteria	**100.0**	0.0	0.0
B5	Bacteria	**100.0**	0.0	0.0
B6	Bacteria	**100.0**	0.0	0.0
B7	Bacteria	**100.0**	0.0	0.0
B8	Bacteria	**100.0**	0.0	0.0
B9	Bacteria	**100.0**	0.0	0.0
C1	Microalgae	0.0	**99.6**	0.4
C2	Microalgae	0.0	**100.0**	0.0
C3	Microalgae	0.0	**99.3**	0.7
C4	Microalgae	0.0	**100.0**	0.0
D1	Microalgae	0.0	**100.0**	0.0
D2	Microalgae	0.0	**100.0**	0.0
D3	Microalgae	0.3	**97.6**	2.1
D4	Microalgae	0.0	**100.0**	0.0
D5	Microalgae	0.0	**100.0**	0.0
H1	Microalgae	0.0	**97.0**	3.0
H2	Microalgae	0.0	**100.0**	0.0
H3	Microalgae	0.0	**99.5**	0.5
H4	Microalgae	0.0	**100.0**	0.0
K1	Microalgae	0.0	**99.9**	0.1
K2	Microalgae	0.0	**100.0**	0.0
K3	Microalgae	0.0	**100.0**	0.0
K4	Microalgae	0.0	**100.0**	0.0
K5	Microalgae	0.0	**100.0**	0.0
K6	Microalgae	0.0	**100.0**	0.0
N1	Microalgae	0.0	**99.9**	0.1
N2	Microalgae	0.0	**99.7**	0.3
N3	Microalgae	0.0	**99.8**	0.2
X1	Microalgae	0.0	**100.0**	0.0
X2	Microalgae	0.0	**100.0**	0.0
X3	Microalgae	0.0	**100.0**	0.0
X4	Microalgae	0.0	**100.0**	0.0
Y1	Microalgae	0.0	**100.0**	0.0

T1	Plants	0.0	0.0	**100.0**
T10	Plants	0.0	0.0	**100.0**
T11	Plants	0.0	0.0	**100.0**
T12	Plants	0.0	0.0	**100.0**
T2	Plants	0.0	0.0	**100.0**
T3	Plants	0.0	0.0	**100.0**
T4	Plants	0.0	0.0	**100.0**
T5	Plants	0.0	0.2	**99.8**
T6	Plants	0.0	0.0	**100.0**
T7	Plants	0.0	0.0	**100.0**
T8	Plants	0.0	0.0	**100.0**
T9	Plants	0.0	0.0	**100.0**

Probability of essential amino acid origins of *Daphnia*, seston, and soils.

ID	Bacteria	Microalgae	Plants
dL1	0.1	**99.6**	0.4
dL2	12.7	**84.0**	3.3
dL3	1.7	**97.6**	0.6
dL4	30.7	**57.3**	12.0
dL5	14.2	**81.6**	4.2
pL2	0.0	**96.8**	3.1
sL1	0.0	0.0	**100.0**
sL2	0.7	2.3	**97.0**
sL4	0.3	0.3	**99.4**
sL5	13.2	18.1	**68.7**

Supporting Table S8 (for Figure 5b)

Linear discriminant analysis output for Figure 5b. According to the MANOVA test, Bacteria, Fungi and Microalgae are significantly different: Pillai Trace=1.71, $F_{10,84}$=50.4, P<0.001.

Coefficients of linear discriminants.

	LD1	LD2
Ile	-0.20	-0.09
Leu	0.56	0.75
Lys	0.69	-0.30
Phe	-0.21	0.09
Thr	-0.07	-0.12
Val	-0.75	-0.40

Proportion of trace.

LD1	LD2
0.61	0.39

Posterior probabilities of the classifier samples.

ID	Actual	Predicted (% probability)		
		Bacteria	Fungi	Microalgae
B1	Bacteria	**99.9**	0.0	0.1
B10	Bacteria	**100.0**	0.0	0.0
B11	Bacteria	**100.0**	0.0	0.0
B12	Bacteria	**100.0**	0.0	0.0
B2	Bacteria	**100.0**	0.0	0.0
B3	Bacteria	**100.0**	0.0	0.0
B4	Bacteria	**100.0**	0.0	0.0
B5	Bacteria	**99.9**	0.0	0.1
B6	Bacteria	**100.0**	0.0	0.0
B7	Bacteria	**99.9**	0.0	0.1
B8	Bacteria	**100.0**	0.0	0.0
B9	Bacteria	**100.0**	0.0	0.0
F1	Fungi	0.0	**100.0**	0.0
F2	Fungi	0.0	**100.0**	0.0
F3	Fungi	0.0	**100.0**	0.0
F4	Fungi	0.0	**100.0**	0.0
F5	Fungi	0.0	**100.0**	0.0
F6	Fungi	0.0	**100.0**	0.0
F7	Fungi	0.0	**100.0**	0.0
F8	Fungi	0.0	**100.0**	0.0
F9	Fungi	0.0	**100.0**	0.0
C1	Microalgae	0.0	0.0	**100.0**
C2	Microalgae	0.0	0.0	**100.0**
C3	Microalgae	0.0	0.0	**100.0**
C4	Microalgae	0.0	0.0	**100.0**
D1	Microalgae	0.0	0.0	**100.0**
D2	Microalgae	0.0	0.0	**100.0**
D3	Microalgae	0.1	0.0	**99.9**
D4	Microalgae	0.0	0.0	**100.0**
D5	Microalgae	0.0	0.0	**100.0**
H1	Microalgae	0.0	0.0	**100.0**
H2	Microalgae	0.0	0.0	**100.0**
H3	Microalgae	0.0	0.0	**100.0**
H4	Microalgae	0.0	0.0	**100.0**
K1	Microalgae	0.0	0.0	**100.0**
K2	Microalgae	0.0	0.0	**100.0**
K3	Microalgae	0.0	0.0	**100.0**
K4	Microalgae	0.0	0.0	**100.0**
K5	Microalgae	0.0	0.0	**100.0**

K6	Microalgae	0.0	0.0	**100.0**
N1	Microalgae	0.0	0.0	**100.0**
N2	Microalgae	0.0	0.0	**100.0**
N3	Microalgae	0.0	0.0	**100.0**
X1	Microalgae	0.0	0.0	**100.0**
X2	Microalgae	0.0	0.0	**100.0**
X3	Microalgae	0.0	0.0	**100.0**
X4	Microalgae	0.0	0.0	**100.0**
Y1	Microalgae	0.0	0.0	**100.0**

Probability of essential amino acid origins in fish.

	Predicted (% probability)		
Sample	Bacteria	Fungi	Microalgae
ch	0	0	100.0
lg	0	0	100.0
xg	0	0	100.0

Supporting Table S8 (for Figure 5c)

Linear discriminant analysis output for Figure 5c. According to the MANOVA test, Bacteria, Microalgae, and Phaeophyceae are significantly different: Pillai Trace=1.11, $F_{10,88}$=11.0, P<0.001.

Coefficients of linear discriminants.

	LD1	LD2
Ile	0.04	-0.48
Leu	-0.80	0.40
Lys	0.37	0.24
Phe	-0.14	-0.01
Thr	0.09	0.19
Val	0.56	-0.17

Proportion of trace.

LD1	LD2
0.92	0.08

Posterior probabilities of the classifier samples.

ID	Actual	Predicted (% probability)		
		Bacteria	Microalgae	Phaeophyceae
B1	Bacteria	**99.6**	0.4	0.0
B10	Bacteria	**100.0**	0.0	0.0
B11	Bacteria	**100.0**	0.0	0.0
B12	Bacteria	**100.0**	0.0	0.0
B2	Bacteria	**100.0**	0.0	0.0
B3	Bacteria	**100.0**	0.0	0.0
B4	Bacteria	**100.0**	0.0	0.0
B5	Bacteria	**99.9**	0.1	0.0
B6	Bacteria	**100.0**	0.0	0.0
B7	Bacteria	**100.0**	0.0	0.0
B8	Bacteria	**100.0**	0.0	0.0
B9	Bacteria	**100.0**	0.0	0.0
C1	Microalgae	0.0	**51.4**	48.6
C2	Microalgae	0.0	**100.0**	0.0
C3	Microalgae	0.0	**96.6**	3.4
C4	Microalgae	0.0	**97.1**	2.9
D1	Microalgae	0.0	**92.2**	7.8
D2	Microalgae	0.0	**98.8**	1.2
D3	Microalgae	0.1	**99.8**	0.1
D4	Microalgae	0.0	**100.0**	0.0
D5	Microalgae	0.0	**95.8**	4.2
H1	Microalgae	0.0	**99.8**	0.2
H2	Microalgae	0.0	**96.5**	3.5
H3	Microalgae	0.0	**97.3**	2.7
H4	Microalgae	0.0	**99.6**	0.4
K1	Microalgae	0.0	**96.7**	3.3
K2	Microalgae	0.0	**80.7**	19.3
K3	Microalgae	0.0	**90.3**	9.7
K4	Microalgae	0.0	**96.0**	4.0
K5	Microalgae	0.0	**98.8**	1.2
X1	Microalgae	0.0	**95.1**	4.9
X2	Microalgae	0.0	**97.5**	2.5
X3	Microalgae	0.0	**99.8**	0.1
X4	Microalgae	0.0	15.7	**84.3**
Y1	Microalgae	0.0	**99.6**	0.4
K6	Microalgae	0.0	**99.8**	0.2
N1	Microalgae	0.0	**99.7**	0.3

N2	Microalgae	0.0	**99.9**	0.1
N3	Microalgae	0.0	**99.9**	0.1
P1	Phaeophyceae	0.0	18.0	**82.0**
P10	Phaeophyceae	0.0	0.3	**99.7**
P11	Phaeophyceae	0.0	34.8	**65.2**
P12	Phaeophyceae	0.0	34.2	**65.8**
P2	Phaeophyceae	0.0	3.6	**96.4**
P3	Phaeophyceae	0.0	25.0	**75.0**
P4	Phaeophyceae	0.0	2.5	**97.5**
P6	Phaeophyceae	0.0	35.9	**64.1**
P7	Phaeophyceae	0.0	2.8	**97.2**
P8	Phaeophyceae	0.0	0.1	**99.9**
P9	Phaeophyceae	0.0	35.8	**64.2**

Probability of essential amino acid origins of mussels.

	Predicted (% probability)		
Sample	Bacteria	Microalgae	Phaeophyceae
gav	0	99.8	0.2
sc	0	99.7	0.3
