# Supplementary material for: Tracing Carbon Sources through Aquatic and Terrestrial Food Webs Using Amino Acid Stable Isotope Fingerprinting
Source: PLoS One. 2013 Sep 17;8(9):e73441. doi: 10.1371/journal.pone.0073441 (PMC3775739; doi:10.1371/journal.pone.0073441)
Supplement: Appendix S1 — FRUITS mixing model data ( Figure 6 ). (PDF) [file pone.0073441.s010.pdf]

Supporting Information Appendix S1

Input data (mean ± standard deviation) of the California mussel (*Mytilus californianus)* for the FRUITS mixing model.

Consumer values:

	$\delta^{13}C_{Val}$	$\delta^{13}C_{Leu}$	$\delta^{13}C_{Lys}$
Mussels (n=2)	-5.53±0.12	-7.43±0.11	3.15±0.43

Food isotope values:

	$\delta^{13}C_{Val}$	$\delta^{13}C_{Leu}$	$\delta^{13}C_{Lys}$
Macrocystis (n=5)	-4.31±0.63	-8.60±0.73	3.02±0.46
Microalgae (n=27)	-4.87±1.33	-7.76±1.85	2.67±1.03
Bacteria (n=12)	-2.37±1.35	-0.76±1.14	0.82±1.18

Food biochemical composition (relative proportions):

	Val	Leu	Lys
Macrocystis (n=1) [8]*	0.42±0.04	0.35±0.03	0.24±0.02
Microalgae (n=14) [9]	0.31±0.01	0.40±0.02	0.29±0.02
Bacteria (n=63) [10]	0.34±0.07	0.39±0.11	0.27±0.13

*Composition derived from one sample; hence, the standard deviation was set to an arbitrary value of 10% of the mean.

Prior information for ranking the most likely food sources was derived from the linear discriminant function analysis (Figure 5c):

[Microalgae]>[Bacteria]
[Microalgae]>[Macrocystis]
[Macrocystis]>[Bacteria]

Output data (relative proportions; mean, standard deviation (Stdev), 2.5, 50 and 97.5 percentiles (pc)) from the FRUITS mixing model.

Estimates on food intake:

	Mean	Stdev	2.5 pc	50 pc	97.5 pc
Macrocystis	0.26	0.11	0.05	0.26	0.46
Microalgae	0.66	0.13	0.45	0.65	0.93
Bacteria	0.08	0.06	0.00	0.06	0.20

Estimates on fraction contribution:

Amino acid	Mean	Stdev	2.5 pc	50 pc	97.5 pc
Val	0.34	0.02	0.30	0.34	0.37
Leu	0.39	0.02	0.36	0.39	0.42
Lys	0.28	0.02	0.24	0.28	0.31

Estimates on food contribution:

Proxy	Food	Mean	Stdev	2.5 pc	50 pc	97.5 pc
$\delta^{13}C_{Val}$	Macrocystis	0.32	0.13	0.07	0.32	0.54
	Microalgae	0.61	0.14	0.39	0.60	0.91
	Bacteria	0.07	0.06	0.00	0.06	0.21
$\delta^{13}C_{Leu}$	Macrocystis	0.24	0.11	0.04	0.23	0.43
	Microalgae	0.69	0.13	0.47	0.68	0.94
	Bacteria	0.07	0.06	0.00	0.06	0.21
$\delta^{13}C_{Lys}$	Macrocystis	0.23	0.11	0.04	0.22	0.42
	Microalgae	0.70	0.13	0.49	0.69	0.94
	Bacteria	0.07	0.06	0.00	0.05	0.23

Appendix S1 (continued).

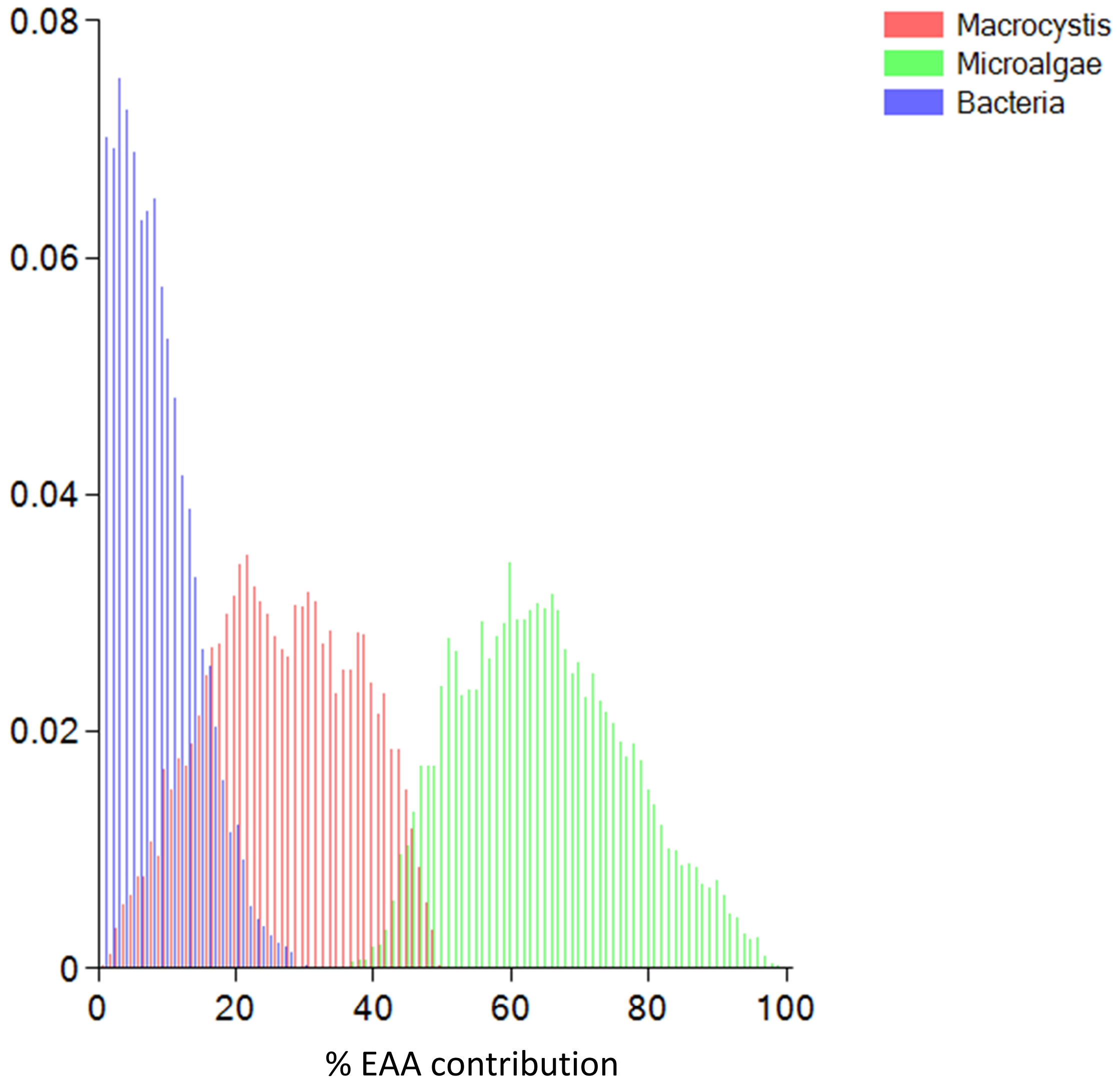

Plot of probability distributions generated with the FRUITS mixing model. The model display the distribution of three potential food sources for the California mussel based on $\delta^{13}C$ values and relative composition of the essential amino acids (EAA); Val, Leu and Lys.

Appendix S1 references

1. Mateus H, Regenstein JM, Baker RC (1976) The amino acid composition of the marine brown alga *Macrocystis pyrifera* from Baja California, Mexico. Botanica Marina. pp. 155-156.
2. Brown MR (1991) The amino-acid and sugar composition of 16 species of microalgae used in mariculture. Journal of Experimental Marine Biology and Ecology 145: 79-99.
3. Simon M, Azam F (1989) Protein content and protein synthesis rates of planktonic marine bacteria. Marine Ecology Progress Series 51: 201-213.
